# Region-Specific Sourcing of Lignocellulose Residues as Renewable Feedstocks for a Net-Zero Chemical Industry

**DOI:** 10.1021/acs.est.4c03005

**Published:** 2024-07-25

**Authors:** Jing Huo, Zhanyun Wang, Pekka Lauri, Juan D. Medrano-García, Gonzalo Guillén-Gosálbez, Stefanie Hellweg

**Affiliations:** † Chair of Ecological Systems Design, Institute of Environmental Engineering, ETH Zürich, 8093 Zürich, Switzerland; ‡ National Centre of Competence in Research (NCCR) Catalysis, ETH Zürich, 8093 Zürich, Switzerland; § Empa-Swiss Federal Laboratories for Materials Science and Technology, Technology and Society Laboratory, 9014 St. Gallen, Switzerland; ∥ International Institute for Applied Systems Analysis (IIASA), A-2361 Laxenburg, Austria; ⊥ Institute for Chemical and Bioengineering, Department of Chemistry and Applied Biosciences, ETH Zürich, 8093 Zürich, Switzerland

**Keywords:** lignocellulose residues, net-zero transition, biomass utilization, chemical industry transition, renewable feedstocks, biobased plastics, life-cycle assessment, biomass availability

## Abstract

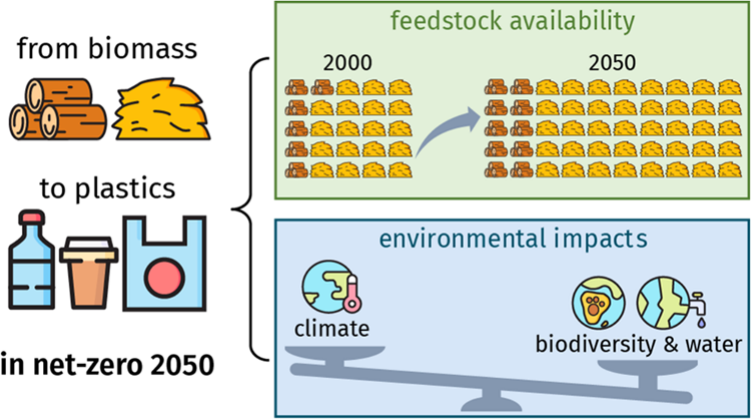

Biobased chemicals, crucial for the net-zero chemical industry, rely on lignocellulose residues as a major feedstock. However, its availability and environmental impacts vary greatly across regions. By 2050, we estimate that 3.0–5.2 Gt of these residues will be available from the global forest and agricultural sectors, with key contributions from Brazil, China, India, and the United States. This supply satisfies the growing global feedstock demands for plastics when used efficiently. Forest residues have 84% lower climate change impacts than agricultural residues on average globally but double the land-use-related biodiversity loss. Biobased plastics may reduce climate change impacts relative to fossil-based alternatives but are insufficient to fulfill net-zero targets. In addition, they pose greater challenges in terms of biodiversity loss and water stress. Avoiding feedstock sourcing from biodiversity-rich areas could halve lignocellulose residues-related biodiversity loss without significantly compromising availability. Improvements in region-specific feedstock sourcing, agricultural management and biomass utilization technologies are warranted for transitioning toward a sustainable chemical industry.

## Abstract 2

This study provides regional sourcing strategies of agricultural and forest residues as renewable feedstocks for chemicals based on their future availability and environmental impacts.

## Introduction

Climate change, biodiversity loss and pollution constitute a triple planetary crisis that demands urgent global action.^1^ The global consensus has now repeatedly underscored the urgency to limit temperature increases to below 1.5 °C, as exemplified by the Paris Agreement,^2−4^ by limiting the extraction and use of fossil fuels—the primary driver of climate change.^5,6^ Fueled by such urgency, the bioeconomy has emerged as a rising alternative approach that promotes the utilization of bioresources to produce goods, energy, and services.^7,8^

The chemical industry is the third-largest emitter of greenhouse gases (GHGs) and the largest industrial consumer of fossil fuels.^9^ In addition to transitioning to renewable energy, the chemical industry also needs to shift from fossil-based feedstocks to renewable feedstocks, including biomass.^10^ Studies have projected the annual demand for biomass for a global net-zero chemical industry to span from 4 to 100 EJ.^11−16^

However, substantial uncertainty exists regarding future biomass availability, with estimates ranging from <100 to over 1000 EJ/year.^17^ Furthermore, concerns have been raised about the sourcing of biomass (i.e., which biomass to choose), including on food security due to potential competition for land,^18,19^ deforestation driven by cropland expansion,^20,21^ and biodiversity loss due to intensified forest management.^22^ These uncertainties and concerns raise a core question about which and how much biomass may realistically contribute in a sustainable way to the future bioeconomy.

In response to these concerns, the focus has shifted toward lignocellulose biomass as a renewable feedstock,^23−25^ including sustainably harvested wood and agricultural residues, which represent the world’s most abundant inedible biomass. A predominant utilization pathway investigated in the net-zero transition of the chemical industry is gasification to methanol.^11−13,15,16^ Methanol can be further converted into key building blocks such as olefins (ethylene and propylene) and aromatics (benzene, toluene, and xylene),^26^ enabling continued use of existing infrastructure while defossilizing chemical production. However, this pathway has a low stoichiometric biomass utilization efficiency (BUE),^27^ with a large part of the biomass converted into CO_2_ and water. Recent progress has been made in increasing BUE by valorizing all three constituents of lignocellulose biomass—cellulose, hemicellulose, and lignin—into platform chemicals such as glucose and xylose,^28−30^ which can be further transformed into various chemical products, including new biobased chemicals without direct fossil-based counterparts.^31^ However, despite these improvements, the environmental sustainability of biomass feedstocks and biobased chemicals is unclear.

Integrated assessment models (IAMs) and life-cycle assessments (LCAs) can be used to assess the environmental benefits and trade-offs of climate change mitigation options. LCAs are often applied at the product or process level. They require detailed process inventory data, which are often not readily available. In contrast, IAMs are useful for macrolevel analysis and are adept at exploring complex interactions between the environment, economy, and society.^32,33^ However, IAMs typically focus on the energy sector, often overlooking the chemical sector as a potential biomass consumer.^34^ The chemical sector not only contributes to temporal carbon storage but also enables cascading use, where waste biobased chemical products can be used for energy production, thus maximizing resource utilization. Moreover, these system-wide analyses often take oversimplified assumptions for biomass utilization, such as directly assuming net-zero emissions under a decarbonized electricity grid.^15,16^ Additionally, other environmental impacts on land and water are either not addressed,^10,14^ or oversimplified with global average resource consumption/availability data,^11,13,15^ overlooking the significance of regional variabilities in water- and land-use-related impacts.

Here, we present a novel approach for the region-specific sourcing of lignocellulose residues, considering both their availability and environmental impacts, accompanied by an open database of this information on various lignocellulose residues on the country level. We combine LCA and IAMs to project the availability of various lignocellulose residues from today through 2050. Then, we conduct prospective LCAs of more than 700 country-residue combinations to assess their associated region-specific environmental impacts, including climate change impacts, water stress and land-use-related biodiversity loss. Based on the availability and impacts of lignocellulose residues, we present a set of region-specific sourcing strategies. The database aims to bridge data gaps from the supply side, and hence, the potential competition for the demand is not covered by the study. However, we further conducted an LCA case study of biobased plastics produced via different routes. We extend the system boundaries by including also downstream production and end-of-life impacts to discuss the relevance of these life-cycle stages in comparison to feedstock sourcing. Finally, we highlight key lessons for future research and policy actions needed for a biobased chemical industry.

## Methods

### Overarching Study Settings

An overview of the study roadmap is presented in Figure S1. The availability of agricultural and forest residues was assessed at a spatial resolution of 200 km × 200 km at ten-year intervals from 2000 to 2050. For agricultural residues, we focused on eight crop types with the highest production volumes.^35^ In the case of forest residues, our analysis was limited to harvests from managed forests. We excluded short-rotation forest plantations due to their highly uncertain future availability^17^ and their higher environmental impacts compared to managed forests.^36^ A full list of all the residues considered is presented in Table S1.

The prospective assessment was conducted based on the narrative of the Shared Socioeconomic Pathway 2 (SSP2), which represented a moderate development framework.^37^ Furthermore, two representative concentration pathways (RCPs) were considered, namely RCPref and RCP1.9:^38^ RCPref represented a reference scenario without climate change mitigation strategies, whereas RCP1.9 was a high-mitigation scenario aimed at limiting the global temperature increase to within 1.5 °C by 2100. This optimistic scenario was selected due to its alignment with a potential net-zero transition in the chemical industry and an anticipated increase in biomass demand. Conversely, RCPref was also included to represent a worst case of the environmental impacts of lignocellulose residues.

The Global Biosphere Management Model (GLOBIOM), a partial equilibrium economic model that focuses on the agriculture, forest, and bioenergy sectors,^39,40^ was used to model the future availability and environmental impacts of lignocellulose residues. Section S1.1.2 in the Supporting Information (SI) presents a brief introduction of the GLOBIOM model and a full list of the model outputs used in this study for the assessments of availability and associated environmental impact.

### Regional Availability of Lignocellulose Residues

We defined three types of potential for lignocellulose residues, as detailed in Table S5. *Theoretical* potential is based solely on the production quantity and yield of the main products. *Ecological* potential additionally considers the necessity to retain a portion of residues on the field to mitigate soil erosion. *Available* potential accounts for further reductions due to losses and allocations for livestock use. While this study primarily evaluates the potential availability of lignocellulose residues against the projected consumption of these residues by the chemical sector, these resources may also be pertinent to other sectors, such as energy.

For agricultural residues, the *theoretical* potential of the total harvest residues of each crop was calculated by multiplying the crop production (a spatially explicit GLOBIOM model output) by their respective residue-to-product ratios (RPRs). In this study, different forms of crop-specific RPR functions were employed, as summarized in Tables S3–S4. For each country-crop combination, the lower- and higher-end *theoretical* potentials of harvest residues were calculated using the different RPR empirical functions to account for uncertainties. From the *theoretical* potential, it was assumed that 2.5 tonnes of harvest residues per hectare of cropland were needed to prevent wind and water erosion of the land,^41^ with the remaining residues considered *ecological* potential. In the cases where the *theoretical* potential of harvest residues was less than 2.5 tonnes/ha, the *ecological* potential was assumed to be 0. Of the *ecological* potential, 70% was considered as the *available* potential,^42^ with the remainder reserved for use as animal feed and bedding.

In addition to harvest residues, the potential of processing residues from agricultural sectors, such as rice husks and sugar cane bagasse, was also considered. It was assumed that 70% of the *theoretical* potential could be utilized as chemical feedstocks, i.e., the *available* potential, to account for potential losses in the value chain.

Concerning forest residues, logging residues encompassed harvest losses, branches, and stumps. The *theoretical* potential for harvest losses was the difference in volume between the stem wood production and the production of roundwood intended for commercial purposes (both were spatially explicit outputs of the GLOBIOM model).^43^ For more detailed descriptions of the *theoretical* potential calculations of logging residues, see Section S1.2.2 in SI. Then, it was assumed that 50% of this *theoretical* potential could be harnessed as feedstocks for the chemical industry, i.e., the *available* potential, while the remaining portion would be subject to technical and environmental limitations and should be left in the forest.^43^

Additionally, the process residues from the forest sector, including sawdust and wood chips, were direct model outputs from GLOBIOM on the regional level (Figure S2). These data were downscaled to match the spatial resolution of the logging residues (200 km × 200 km), assuming that process residues exhibited the same spatial distribution pattern as logging residues.

### Life-Cycle Inventories of Lignocellulose Residues

The environmental impacts associated with lignocellulose residues were assessed using prospective LCAs, implemented with the Brightway2 framework,^44^ as outlined in Figure S4. To assess system changes in future scenarios, the premise tool (v1.4.1)^45^ that couples ecoinvent 3.8^46^ with IMAGE, an extensively utilized integrated assessment model,^47^ was used to generate background life-cycle inventory (LCI) data sets. For consistency, the same SSP and RCP scenarios were selected for IMAGE as for GLOBIOM. Our cradle-to-gate analysis covers all harvesting activities in croplands and forests. Transportation to downstream users is excluded to provide flexibility for applying the data in future cradle-to-grave LCA studies, enabling tailored analysis of residue applications (our data can be found at Zenodo^48^).

When lignocellulose residues were actively harvested for sale as chemical feedstocks, they would transition from waste to coproducts of the main forest or agricultural products. Therefore, the associated environmental impacts of biomass cultivation and harvesting were allocated to all the coproducts according to their economic value. This economic allocation of impacts captured the rationale behind production—should the demand for residues rise, particularly under the RCP1.9 scenario, then their market value would also increase. An increase in residue value could cause the production process to be more financially appealing, which, in turn, may drive land-use changes.

#### Differentiation of the Land-Use Intensities of Various Land Types

GLOBIOM-forest, a submodel of GLOBIOM that provides a more comprehensive representation of the forest sector, was employed to represent the forest sector, while the GLOBIOM full model was utilized to account for the agricultural sector (see Section S1.4.1 in SI for detailed description of land-use intensities in both models). To facilitate the integration of these models, the land-use data of both models were harmonized. For this purpose, the total forest area (including primary, secondary, and managed forests) was scaled from the GLOBIOM-forest submodel to align with the total forest area (covering unmanaged and managed forests) in the complete GLOBIOM model for each country. This resulted in only minor alterations to the total forest area within the GLOBIOM-forest model, with fluctuations ranging from −6 to +2%, contingent on the specific year and scenario considered.

#### Land-Use Change Associated with Lignocellulose Residues

Following the Guidelines for National Greenhouse Gas Inventories by the Intergovernmental Panel on Climate Change (IPCC),^49^ it was assumed that after a land-use change, the affected land remained in a transitional period for a duration of 20 years. Consequently, the areas dedicated to each land-use type in each country were evaluated for both the reference year and the year 20 years prior to the reference year (e.g., 2050 as the reference year and 2030 as the beginning year of the assessment), and all impacts (including climate change and biodiversity loss) associated with the land-use changes were distributed evenly over these 20 years. Allocation of the land-use changes for cropland was performed following the PAS 2050-1 Guidelines.^50^ A similar allocation process was developed for managed forests with harvesting activities (Figure S5). According to the PAS 2050-1 Guidelines, the impact of the land-use change was exclusively assigned to products associated with an increase in harvest areas during the assessed period. Conversely, products with a reduced harvest area did not receive any allocation of impacts or credits of the land-use changes. For example, in the case of deforestation and land conversion to agricultural land, only crops with land-use expansion in the past 20 years received the impacts of land-use change.

#### Other Regionalized Prospective Life-Cycle Inventory Data (LCIs)

The regionalized prospective LCIs of agricultural production activities were modeled with the reference flow of one hectare of cropland. For a detailed description of the procedures used to create the inputs and emissions, see Table S7. In brief, they were modeled in the following four steps: (1) Country- and crop-specific blue water consumption data were obtained from Pfister et al.^51^ (2) Spatially explicit GLOBIOM model outputs, including the application rates of nitrogen and phosphorus fertilizers for each crop, were averaged at the country level. (3) GHG emissions from land-use changes and direct and indirect onsite emissions from fertilizers and crop residues were calculated based on tier 1 emission factors and constants according to the IPCC Guidelines.^49^ (4) Other inputs and emissions relied on the background data sets provided by Agri-footprint 6, a database known for its extensive coverage and reliability regarding the agricultural sector.^52^

The regionalized LCIs of forest residues were created by updating the energy mix, transportation, land use, and land-use changes. Given the detailed modeling of wood harvesting and processing activities for Switzerland in ecoinvent 3.8, these served as the foundational data sets for creating regionalized LCIs. Activities involving energy and transportation flows were relinked to the regionalized and prospective background LCIs. The products from the managed forest were sawlogs, pulpwood, other industrial wood, fuel wood, and logging residues, in accordance with the structure of the GLOBIOM model. The allocation of land use and land-use changes to these products was determined using economic allocation, based on their respective regional prices determined by the GLOBIOM model. In addition to logging residues, wood chips and sawdust, which are generated as coproducts in sawmills as process residues, were also assessed for their impacts based on economic allocation considering the future prices of sawn wood and process residues (GLOBIOM model output). For a comprehensive list of the updated data sets from ecoinvent 3.8, see Table S8.

### Life-Cycle Impact Assessment of Lignocellulose Residues

The assessed environmental impacts included climate change impacts, water stress, and land-use-related biodiversity loss. Following the Global Guidance for Life Cycle Impact Assessment Indicators by the United Nations Environment Program (UNEP) and the Society of Environmental Toxicology and Chemistry (SETAC),^53^ the climate change impacts were quantified as the global warming potential over 100 years (GWP100). The global temperature change potential over 100 years (GTP100) was in addition used as sensitivity analysis to reflect the long-term effects of temperature change. Water stress was indicated according to the Available WAter REmaining (AWARE) method.^54^ Land-use-related biodiversity loss was quantified with potentially disappeared fractions of species (PDF). The characterization factors were recently updated under the framework of the Global Life Cycle Impact Assessment Method (GLAM) Initiative^55^ and were applied in this study. The mapping of land-use classifications in GLOBIOM (life-cycle inventory) and in this life-cycle impact assessment method can be found in Table S9. For water stress and land-use-related biodiversity loss, country-specific characterization factors were applied to enable regionalized impact assessments.

### Life-Cycle Assessment of Biobased Plastics

A life-cycle assessment case study was performed for biobased polypropylene (PP) and polylactic acid (PLA) under the RCP1.9 scenario. PP and PLA display the same functions in many applications with similar weight; hence, functional unit is 1 kg of plastic.

For PP, the production chain was assumed as follows: (1) Methanol production from the gasification of lignocellulose residues,^26^ (2) propylene production based on methanol-to-olefin technology,^56^ and (3) polymerization into PP.

The PLA production chain was assumed to consist of (1) glucose production from lignocellulose fractionation using aldehyde treatment,^57^ (2) lactic acid production from glucose fermentation, and (3) polymerization into PLA.

The LCI data for glucose were based on a process simulation of lignocellulose fractionation conducted with Aspen Plus v12. A detailed description of the process simulation and LCI can be found in Section S1.5 in SI. This process coproduce platform chemicals including glucose, xylose, and lignin. Considering the unknown future economic value of these products, mass allocation was applied, resulting in equal specific impacts for all coproducts. The LCI data for other processes were sourced from either the literature (methanol and propylene production)^26,56^ or the IHS Markit (lactic acid and polymerization of PP and PLA).^58^ The inventory data are summarized in Tables S14–S16.

## Results and Discussions

### Large Untapped Potential of Lignocellulose Residues

**Figure 1:**
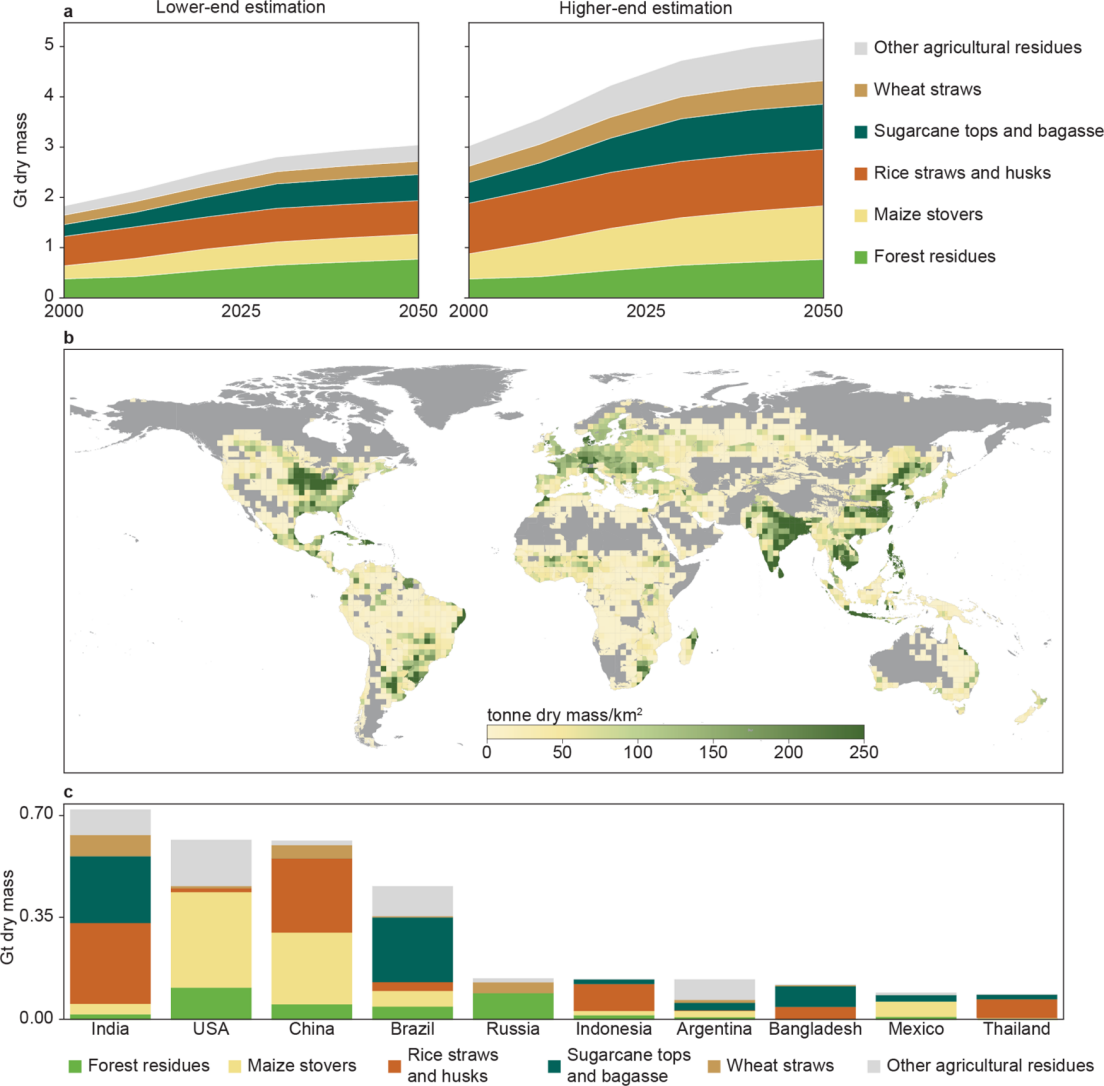
(a) Lower- and higher-end estimations of the global available potential of lignocellulose residues by biomass type from 2000 to 2050. (b) Spatial distribution of lignocellulose residues in 2050 at 200 km × 200 km resolution (the higher-end estimation is shown here; for the lower-end estimation, see Figure S7a). The Gray area reflects no cropland or managed forest in the specific region. (c) Top 10 countries with the highest potential for lignocellulose residues by biomass type in 2050 (the higher-end estimation is shown here; for the lower-end estimation, see Figure S7b).

The projections of the *available* potentials of lignocellulose residues show a promising upward trend, from 1.8–3.0 gigatonnes dry mass (Gt DM/year) in 2000 to 3.0–5.2 Gt DM/year (equivalent to 48–83 EJ/year) in 2050 under the SSP2 framework (Figure 1a). The ranges correspond to the higher- and lower-end estimations, mainly caused by uncertainties in the empirical crop-to-residue ratio functions. Our estimates align with previous studies, which collectively suggest a global residue availability averaging approximately 55 EJ/year by 2050, within a range of 12–76 EJ/year.^59^ Agricultural residues (75–85% of the total potential), particularly residues from maize, rice, and sugar cane, represent major lignocellulose residues.

The geographic distribution of lignocellulose residues is heterogeneous, with more than half of the potential in 2050 projected to be concentrated in India, the United States, China, and Brazil (Figure 1b). The compositions of residues also vary greatly across countries. According to the higher-end estimation in 2050, forest residues account for only 0.4% of residues in India, while they represent a major source of residues in Russia (62%) (Figure 1c). This heterogeneous distribution and the low share of forest residues in the total potential of lignocellulose residues call for research on how to best valorize all different lignocellulose residues, beyond the typical biorefinery focus of wood.^28^

The main value of biomass for chemical production lies in its rich biogenic carbon content.^60^ Lignocellulose residues, with a 50% carbon content,^61^ offer 1.5–2.6 Gt of carbon by 2050. On the demand side, the global plastics market—a major segment of the chemical industry—registered a global demand of 0.46 Gt in 2019,^62^ with expectations to double by 2050.^14^ Considering the carbon content in various plastic types (Table S6), approximately 0.68 Gt of carbon will be needed to match the annual demand for plastic production by 2050. Using the carbon content as a simple proxy for feedstock demands, the potential carbon supply from lignocellulose residues could be more than double the required carbon for the expected plastic production. This highlights that lignocellulose residues, as a substantial yet largely untapped resource, could adequately supply the carbon needs for the growing global demand for plastics.

### Climate Change Impacts Driven by Agriculture

Lignocellulose residues are estimated to have on average 0.11 kg CO_2_-eq/kg DM climate change impacts from cradle (activities from biomass cultivation and harvesting) to gate (the point of delivery to chemical manufacturing sites, excluding the manufacturing process) under the RCP1.9 scenario in 2050.

**Figure 2:**
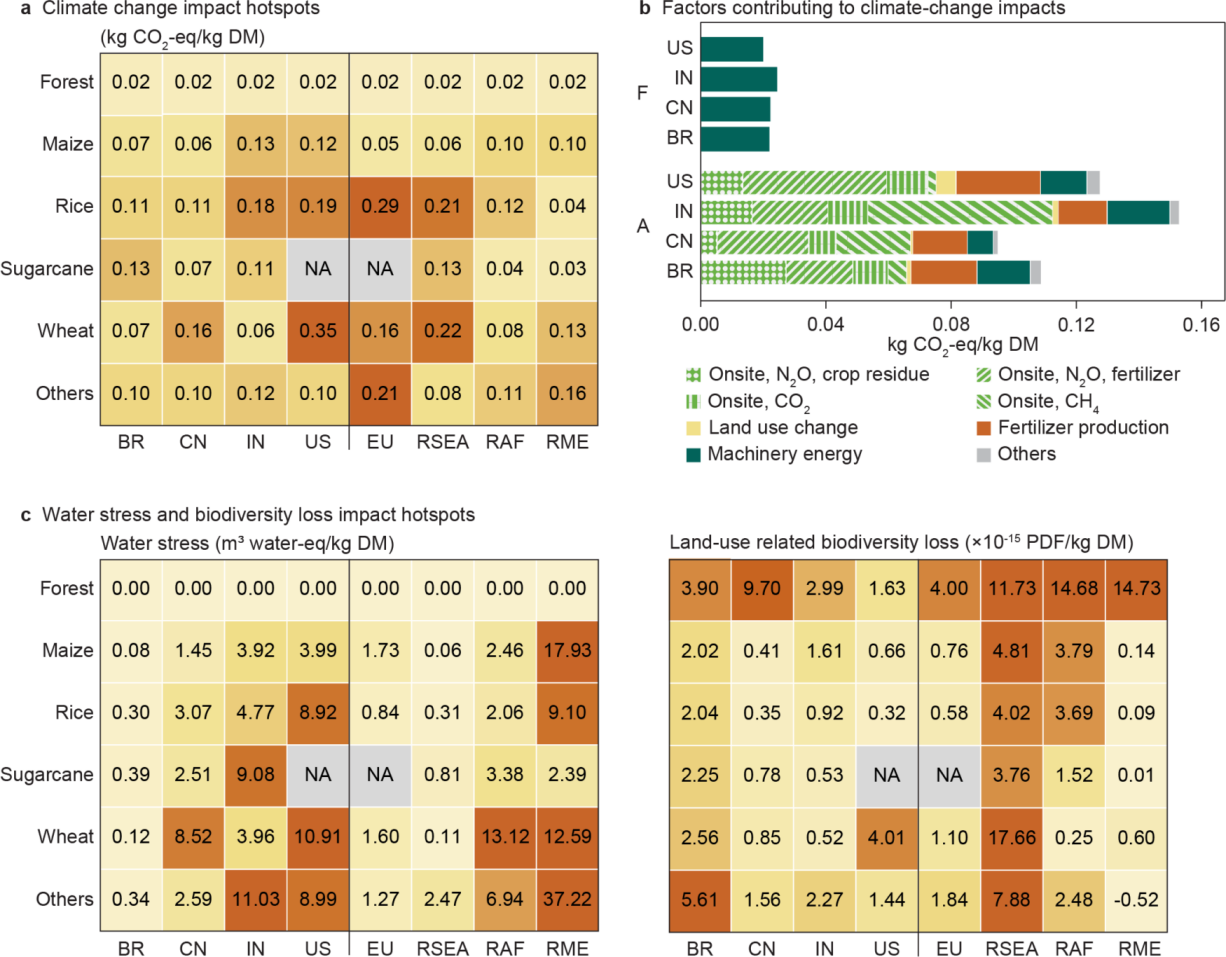
Cradle-to-gate environmental impacts of lignocellulose residues under the RCP1.9 scenario in 2050. (a) Climate change impact hotspots quantified as the global warming potential over 100 years (GWP100) associated with each biomass type in selected countries and regions. (b) Contribution analysis of the climate change impacts (GWP100) in the four countries with the highest lignocellulose residue potential. (c) Water stress and land-use-related biodiversity loss impact hotspots associated with each biomass type in selected countries and regions. Impact distribution across all countries are presented in Figure S9. Impacts under the RCPref scenario are presented in Figures S10–S11. The impacts of agricultural residues are quantified with only harvest residues. The impacts of forest residues are based on the availability-weighted average of both harvest and process residues. Abbreviations: DM, dry mass; F, forest residues; A, agricultural residues; BR, Brazil; CN, China; IN, India; US: the United States of America; EU, the European Union; RSEA: Region South East Asia; RAF, Region Africa; RME, Region Middle East.

The impacts of lignocellulose residues vary depending on their type and source (Figure 2a). Forest residues have comparatively low cradle-to-gate climate change impacts (a global average of 0.020 kg CO_2_-eq/kg DM), which are mainly contributed by machinery energy during wood harvesting. Agricultural residues generally have greater cradle-to-gate impacts than forest residues, ranging from maize stover at 0.091 kg CO_2_-eq/kg DM to barley straw at 0.22 kg CO_2_-eq/kg DM. Predominantly, the impacts come from hard-to-abate onsite GHG emissions during crop cultivation, accounting for 60–74% of the overall impacts in the four leading countries rich in lignocellulose residues (Figure 2b). Nitrous oxide (N_2_O) is the largest contributor and is released from nitrogen fertilizers and the degradation of crop residues that are left on the field to prevent wind and water erosion. Additionally, methane (CH_4_) is emitted from flooded rice fields due to the anaerobic decomposition of organic materials,^49^ with India leading and followed by China (Figure 1c). Rapid development and implementation of advanced technologies and farming practices are necessary to reduce the impacts of climate change on agriculture and, thus, for agricultural residues to be more appealing chemical feedstocks. For example, researchers have demonstrated the possibility of fertilizer production with net-zero GHG emissions,^63,64^ increasing nitrogen use efficiency, and immobilizing nitrogen residues, among others, as a means to mitigate onsite N_2_O emissions.^65,66^ Moreover, alternative rice cultivation methods can reduce CH_4_ emissions from rice fields up to 30%.^67^ Negative emission technologies such as bioenergy with carbon capture and storage and enhanced rock weathering may address the remaining hard-to-abate emissions in the agricultural sector.^67^

### Non-Negligible Water Stress and Land-Use-Related Biodiversity Impacts in Many Regions

Water stress predominantly arises from intensive agricultural irrigation and is negligible for forest residues. Globally, water stress associated with agricultural residues is approximately 4.8 m^3^ water-eq/kg DM on average. It varies particularly widely across regions (Figure 2c) and is subject to the evapotranspiration of crops and the effective precipitation levels in a given region. In the Middle East and North Africa, water stress associated with agricultural residues could reach as high as 30 m^3^ water-eq/kg DM, indicating severe challenges there.

Land-use-related biodiversity loss (Figure 2c), measured in potentially disappeared fractions of species (PDF),^55^ is related to land occupation and transformation. Under the RCP1.9 scenario, an increase in wood harvesting per hectare in 2050 is assumed compared to the baseline in 2030. This intensification of forest management results in more than twice as much biodiversity loss in forest residues than agricultural residues. Land-use-related biodiversity loss has strong regional variations. In some areas, biodiversity might recover when land undergoes transformation from 2030 to 2050, returning to a closer-to-nature state (e.g., when cropland is transformed into managed forests). In contrast, some islands in Southeast Asia are home to numerous globally endangered species, rendering lignocellulose residues from these areas more likely to contribute to greater land-use-related biodiversity loss— exceeding the global average by more than 10-fold.

### Land-Use Change and Management Intensification Resulting in High Impacts in Key Regions

**Figure 3:**
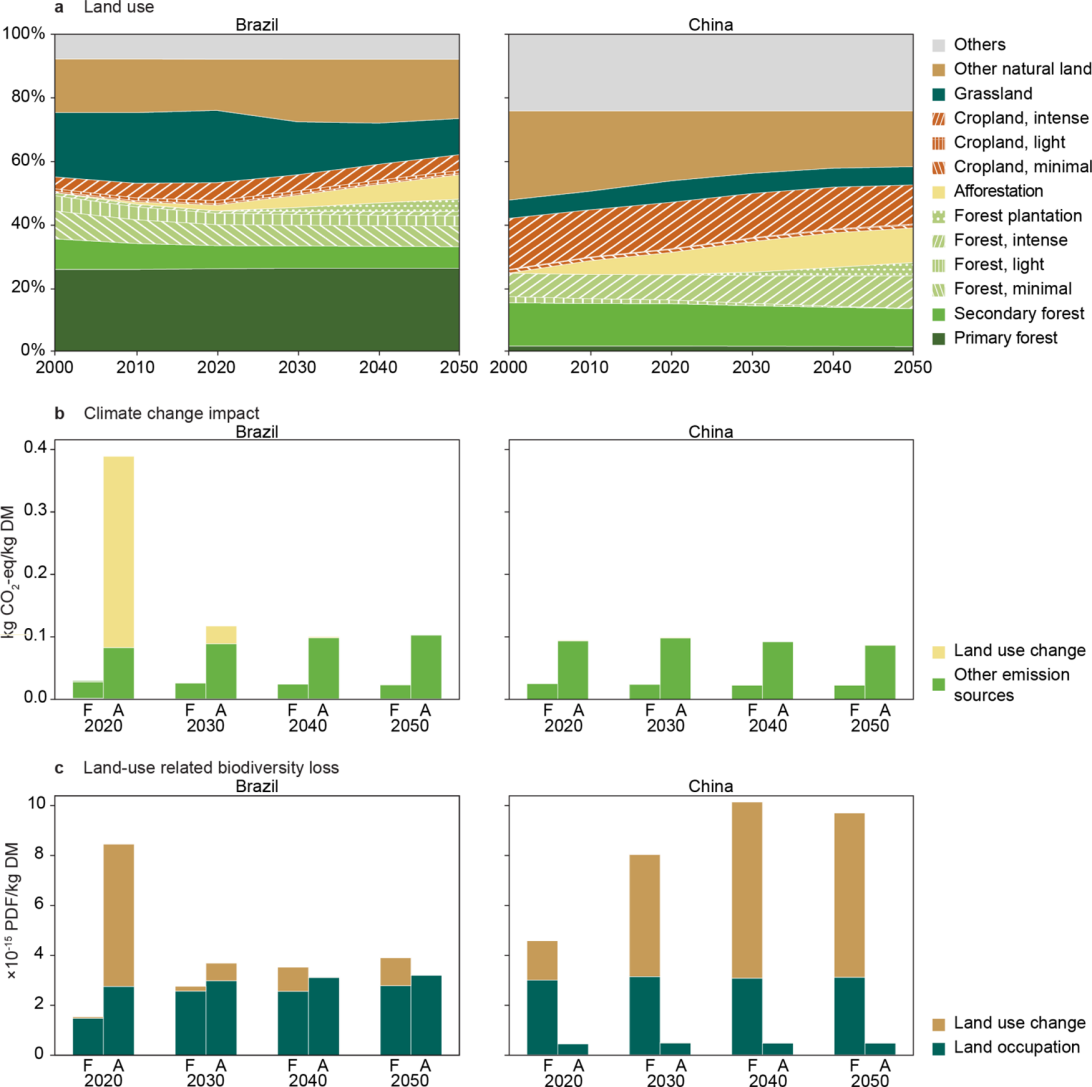
(a) Land-use percentages in Brazil and China under the RCP1.9 scenario. (b, c) Climate change impacts and land-use-related biodiversity loss impacts, respectively, of forest residues and agricultural residues contributed by land-use change (including change in management intensity) and other factors in Brazil and China under the RCP1.9 scenario. For results of other nations and the RCPref scenario, see Figures S12–S15. Other major sources contributing to climate change impacts include onsite emissions, fertilizer production and machinery energy. Abbreviations: F, forest residues; A, agricultural residues.

Land-use change can greatly contribute to both climate change and biodiversity loss. Its impacts are attributed to products harvested from land that have expanded over the 20 years leading up to the assessment year^50^ (e.g., looking at changes from 2030 to 2050 for an impact assessment in 2050). Figure 3 shows the land-use change in Brazil and China over the years and its effect on the impacts of agricultural and forest residues. These two countries serve as two examples of major lignocellulose residue suppliers with opposing trends.

In Brazil, agricultural residues have greater biodiversity loss impacts than in China, due to the presence of more endemic and endangered species. In 2020, Brazil’s agricultural residues showed high climate change and biodiversity loss impacts, with 78 and 67% of these impacts, respectively, attributed to land-use change, largely driven by cropland expansion and associated deforestation in the preceding 20 years. These high impacts warn against land-use change from forests to croplands, especially in biodiversity-rich regions. Meanwhile, Brazil has committed to zero deforestation in the Amazon rainforest by 2030,^68^ which may, if implemented, result in a decrease in land-use-related impacts there (Figure 3b,c).

Conversely, China shows no cropland expansion under the RCP1.9 scenario according to the GLOBIOM results (Figure 3a), resulting in relatively stable land-use-related impacts over time. However, increased wood harvesting from increasing the management intensity of forests (here also marked as land-use change) contributes to rising biodiversity loss impacts associated with forest residues.

### Need for Regionalized Feedstock Sourcing Guided by Availability and Impacts

The chemical industry needs to develop sustainable feedstock-sourcing strategies that navigate the trade-offs between climate benefits and biodiversity loss impacts. Globally, forest residues generally have no water stress impacts and are associated with 84% lower cradle-to-gate climate change impacts than agricultural residues. However, this benefit is counterbalanced by greater land-use-related biodiversity loss—more than double that associated with agricultural residues on a global average.

**Figure 4:**
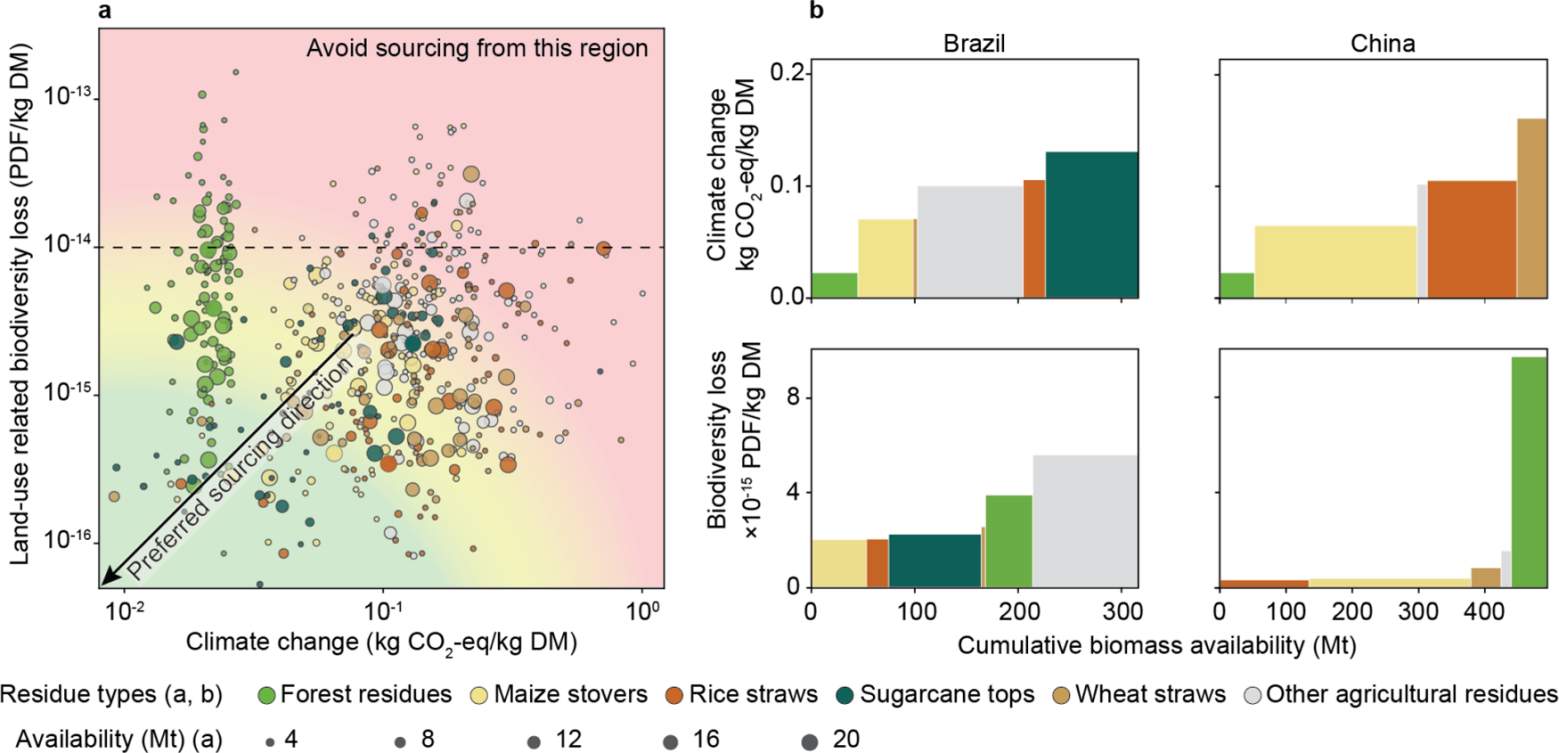
(a) Impact trade-offs between climate change and land-use-related biodiversity loss under the RCP1.9 scenario in 2050. Each circle represents one country-residue combination. For kernel density estimations of impacts for each lignocellulose residue type, refer to Figure S16. (b) Climate change and land-use-related biodiversity loss impact-merit-order curves of lignocellulose feedstocks in Brazil and China in 2050 under the RCP1.9 scenario. For comparisons in India and the United States, as well as water-stress merit-order curves, refer to Figure S17.

Figure 4a presents the trade-offs between climate change and land-use-related biodiversity impacts across residues and countries. The feedstocks in the bottom-left corner of these trade-off graphs are preferred—zones indicating low impacts on both climate and biodiversity. Excluding biomass feedstocks with a biodiversity impact exceeding 10^–14^ PDF/kg DM enables a 43% reduction in total biodiversity loss when leveraging the full available potential of the remaining feedstocks. This strategy only marginally reduces feedstock availability by 5.8%.

Besides the strategy to avoid biomass sourcing from vulnerable ecoregions, we further introduce impact-merit-order curves, depicted in Figure 4b, to guide systematic sourcing at the country level. They rank feedstocks based on environmental impacts against their supply potential, supporting decision-making by stakeholders. For instance, maize stover in both Brazil and China is a feedstock with comparatively low climate change and biodiversity impact. However, feedstocks with low climate change impacts do not invariably correspond with low biodiversity loss impacts. Forest residues in China, for example, exhibit lower climate footprints, but nearly ten times greater biodiversity loss impacts than most agricultural residues. These high biodiversity loss impacts come from the projected increase in demand for forest products, which leads to more intensive forest management practices.

### Insufficient Climate Benefits of Biobased Plastics from Shifting Feedstock Alone

In addition to biomass feedstocks, the environmental impacts of biobased plastics also depend on the production and end-of-life treatment. Therefore, we expand the system boundaries to investigate the environmental sustainability of biobased plastic value chains.

Biomass feedstock consumption and the impacts of biobased plastics vary with the utilization pathways. Polypropylene (PP) production via biomass gasification and methanol-to-olefin processes is biomass intensive, consuming 6.8 kg of biomass per kg of plastics. Under this biomass-intensive method, meeting the projected plastic demand by 2050 would need 6.8 Gt (110 EJ) lignocellulose residue, surpassing its *available* potential. In contrast, compared with that of PP, polylactic acid (PLA) production using propionaldehyde fractionation and glucose fermentation is markedly more biomass efficient,^57^ reducing biomass consumption by 80%.

**Figure 5:**
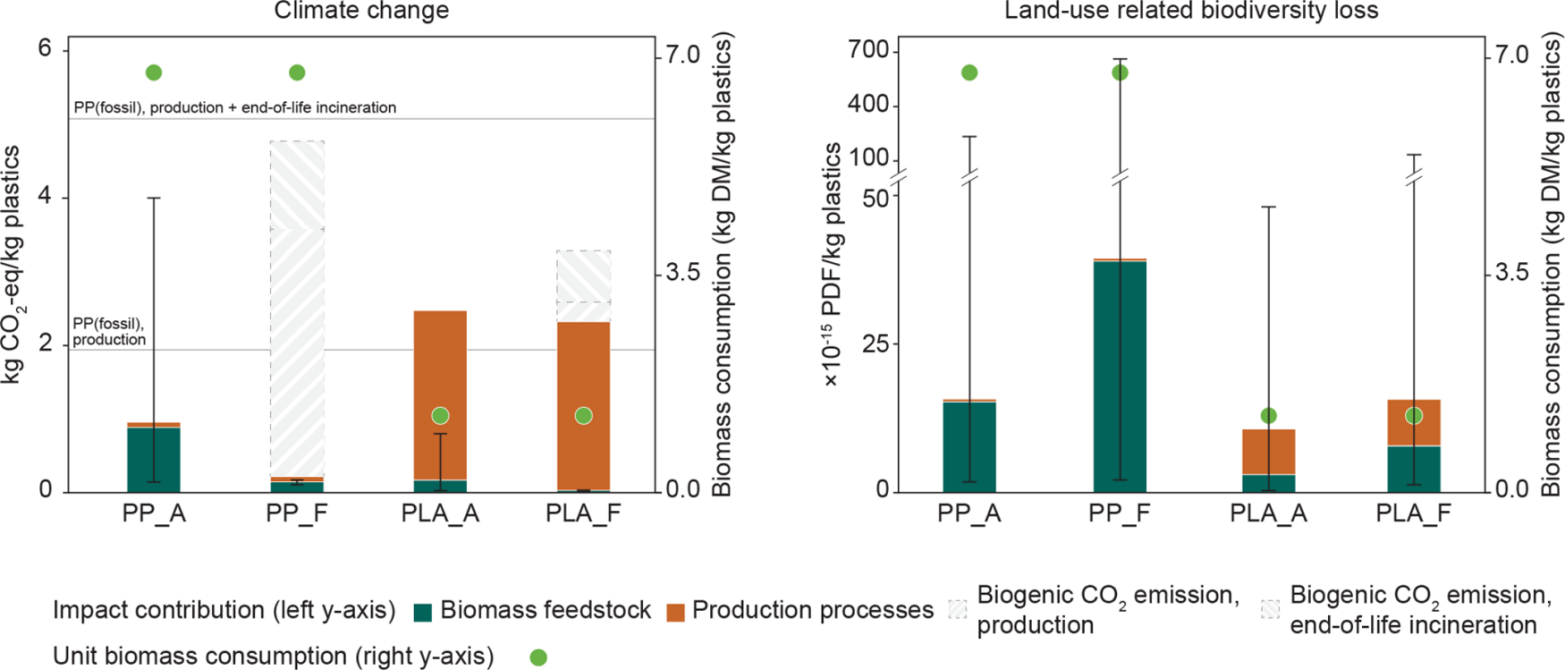
Climate change impacts and land-use-related biodiversity loss impacts of the production and end-of-life incineration of 1 kg biobased plastics in 2050 under the RCP1.9 scenario. The global average impacts of agricultural and forest residues are presented, with error bars representing the range of impacts from biomass feedstock at the 2.5 and 97.5% quantiles. The green dots represent the unit biomass consumption, as indicated on the right *y*-axis. “Biogenic CO_2_ emissions, production” refers to the climate change impacts caused by direct biogenic CO_2_ emissions from the manufacturing process, e.g., the gasification of biomass for methanol production. “Biogenic CO_2_ emissions, end-of-life incineration” refers to the climate change impacts caused by biogenic CO_2_ emissions from the incineration of waste plastics. Abbreviations: PP_A: polypropylene from agricultural residues; PP_F: polypropylene from forest residues; PLA_A: polylactic acid from agricultural residues; PLA_F: polylactic acid from forest residues.

Compared with their fossil-based counterparts, biobased plastics can have lower climate change impacts (Figure 5, left). Specifically, PP made from agricultural residues shows a 51% reduction in climate change impacts during production and an 81% reduction, including end-of-life incineration. However, these reductions fall short of the industry’s net-zero target to reduce 95% of emissions by 2050.^3^ The feedstock choice significantly influences the climate change impacts of PP, highlighting the importance of low-impact feedstock sourcing. Moreover, the laboratory-scale biomass fractionation technique investigated in this study (see Section S2.7 in SI for LCA results) leads to greater climate change impacts of PLA. However, with end-of-life incineration considered, PLA outperforms fossil-based PP, and with technological advancements, its impacts are poised for further reduction.

Biogenic CO_2_ is emitted during the production chain and end-of-life incineration of biobased plastics. The climate change impacts of these emissions depend on the rotation period of the biomass and the storage period of biogenic carbon.^69,70^ Biogenic CO_2_ released from agricultural residues is sequestered again by the regrowth of biomass within a short amount of time. In contrast, biogenic CO_2_ released from forest residues may contribute to climate change due to the long rotation period and delayed CO_2_ resequestration, and this impact is also influenced by forest management practices (i.e., clear-cut vs selective harvesting).^71^ Worst-case scenarios, as depicted in Figure 5 (left), show that biogenic CO_2_ is the predominant source of emissions for forest residue-based PP. However, this result does not imply that forests should be transformed into cropland because the impacts from land-use change can be enormous (e.g., agricultural residues in Brazil in 2020, as shown in Figure 3). Instead, effective mitigation of end-of-life climate change impacts could be achieved through strategies such as the promotion of durable and cascading use of biobased products (e.g., valorizing waste wood from the construction sector as feedstocks for chemicals with long lifespans^72^), and carbon capture technologies for incineration processes.

Land-use-related biodiversity loss is almost entirely contributed by biomass, including feedstock sourcing and bioenergy use, in the projected decarbonized energy system (Figure 5, right). This impact is negligible for fossil-based PP but much greater for biobased PP due to its high biomass consumption. Biomass sourced from regions with high biodiversity can exponentially increase this impact, necessitating a strategy to avoid biomass sourcing in vulnerable ecoregions.

Region-specific biomass feedstock sourcing and efficient utilization of lignocellulose residues are key to managing feedstock demand and mitigating biodiversity trade-offs. Prioritizing utilization routes with high biomass utilization efficiency and improving technology scale-up and optimization are essential steps toward a low-impact biobased chemical industry.

### Model Uncertainties and Limitations

Our strategies for sourcing lignocellulose residues are subject to some uncertainties and limitations. First, the *available* potential will be affected by the variability of future demand for crops and wood products that may deviate from the SSP2 framework as modeled here, impacting sourcing decisions. Second, with economic allocation of impacts, we assume the increasing demand of lignocellulose residues with competitive use from other sectors may result in land-use change. However, the future prices of lignocellulose residues are also uncertain. A lower residue price would lead to less impact allocated to lignocellulose residues. Third, crop residues are not endogenously included in GLOBIOM as a potential resource to satisfy the biomass demand depicted by the SSP and RCP scenarios. This setting could influence future land-use patterns, e.g., more land is transformed for short-rotation plantations. These uncertainties are addressed by sensitivity analysis (Section S3.1 in SI).

Additionally, we focus on the cradle-to-gate impacts of lignocellulose feedstocks, with only one case study including downstream production and end-of-life stages for plastics. The complexity of the biobased chemical production chain may lead to greater impacts of biobased chemicals than of their fossil-based counterparts. The climate change impacts of released biogenic CO_2_ emissions are only discussed with one worst-case scenario analysis due to the absence of standardized methods. In addition, while the climate change impacts of land-use change from forest to agricultural land is quantified in this study, this is not the case for intensified land management. Intensified forest management, for example, may lead to a decline in biodiversity loss, as shown in the study, and may decrease the carbon stock in the forest with an impact on climate change, which is not quantified following the IPCC guidelines.^49^ Likewise, additional removal of lignocellulose residues may pose potential climate-change impacts through the reduction of soil organic carbon^73−75^ and biodiversity loss due to habitat disruption.^76^ These specific impacts are not quantified in this study due to the absence of standardized assessment methods, but warrant future research. For a comprehensive analysis of these limitations, see Section S3.2 in SI.

### Implications

In this study, we provide a holistic, region-specific approach to sourcing lignocellulose residues as feedstocks for a future net-zero chemical industry, considering both their availability and associated environmental impacts. Our results highlight the following key lessons for future research and policy action.

First, efficient utilization of lignocellulose residues is crucial, as the available supply is not much higher than the demand from the chemical sector, given that there are process losses and potential future competing demands from other sectors such as energy. Future research should focus on the competitive use of these resources to determine the most environmentally beneficial uses of biomass. Furthermore, the widespread and dispersed distribution of lignocellulose residues underscores the need for region-specific strategies tailored to unlock the full *available* potential of lignocellulose residues after discounting for residues that should remain in the forest or on agricultural land for ecological reasons.

Second, biobased chemicals can reduce climate change impacts compared to their fossil-based counterparts, emphasizing the need for upscaling their production processes for higher raw material and energy efficiency. The selection of biomass feedstocks is critical, as the climate change impacts of biobased chemicals heavily depend on the type of lignocellulose residues used. With agricultural residues forming a crucial portion of these residues, the realization of a net-zero biobased chemical industry hinges upon the successful achievement of net-zero agriculture. This encompasses the prevention of deforestation for agricultural expansion and an increased focus on research and transitioning into sustainable agricultural practices, especially improvements in mineral fertilizer use and mitigation of onsite GHG emissions. Moreover, improved forest management practices (e.g., selective harvesting instead of clear-cutting) can reduce biodiversity impacts and potentially increase carbon storage in forest systems, thereby mitigating climate change impacts. Further reductions in climate change impacts of the chemical industry depend on energy system decarbonization and improved end-of-life strategies for chemical products, such as capturing carbon during plastic incineration.

Third, the biodiversity loss and water stress impacts of biobased chemicals are mainly driven by the sourcing of biomass feedstocks. Our LCA results reveal significant regional differences in associated water stress and land-use-related biodiversity loss. The trade-offs between climate benefits, water stress and biodiversity loss impacts need to be addressed on regional basis. Biomass harvesting from biodiversity hotspots should be avoided to prevent burden shifting. Environmental impact-merit-order curves and impact trade-off plots (Figure 4) can help decision makers identify sustainable strategies for sourcing biomass feedstocks.

Achieving a net-zero chemical industry requires the integration of various technologies, including the utilization of renewable feedstocks such as CO_2_ and biomass, increased circularity, the use of low-carbon energy, and carbon capture and utilization.^77^ It is crucial to have an in-depth understanding of the feasibility of each pathway involved. We conclude that the potential use of lignocellulose residues for a low-carbon chemical industry is crucial, yet careful management of resources and technology improvement of biomass utilization pathways are essential prerequisites for the sustainability of this transition.

## Data Availability

The following data generated in this study are available at https://zenodo.org/doi/10.5281/zenodo.12591834. • Data 1–data sets presenting the *theoretical*, *ecological*, and *available* potential of various lignocellulose residues on the GLOBIOM grid level (200 km × 200 km). • Data 2–data sets presenting the *theoretical*, *ecological*, and *available* potential of various lignocellulose residues on the country level and their corresponding climate-change impacts, water stress, and land-use-related biodiversity loss impacts

## Supporting Information Available

Details on methods, additional results and discussions (PDF)
