# Supplementary material for: Region-Specific Sourcing of Lignocellulose Residues as Renewable Feedstocks for a Net-Zero Chemical Industry
Source: Environ Sci Technol. 2024 Jul 25;58(31):13748–59. doi: 10.1021/acs.est.4c03005 (PMC11308523; doi:10.1021/acs.est.4c03005)
Supplement: Supplementary file 1 — es4c03005_si_001.pdf [file es4c03005_si_001.pdf]

Supporting Information

Region-specific sourcing of lignocellulose residues as renewable feedstocks for a net-zero chemical industry

Jing Huo*[1,2], Zhanyun Wang[2,3], Pekka Lauri[4], Juan D. Medrano-García[2,5], Gonzalo Guillén-Gosálbez[2,5] and Stefanie Hellweg[1,2]

Author Information:

[1]Chair of Ecological Systems Design, Institute of Environmental Engineering, ETH Zürich, 8093 Zürich, Switzerland.

[2]National Centre of Competence in Research (NCCR) Catalysis, ETH Zürich, 8093 Zürich, Switzerland

[3]Empa-Swiss Federal Laboratories for Materials Science and Technology, Technology and Society Laboratory, 9014 St. Gallen, Switzerland

[4]International Institute for Applied Systems Analysis (IIASA), A-2361 Laxenburg, Austria

[5]Institute for Chemical and Bioengineering, Department of Chemistry and Applied Biosciences, ETH Zürich, 8093 Zürich, Switzerland

Correspondence and requests for materials should be addressed to Jing Huo: jhuo@ethz.ch

This supporting information contains 49 pages, 21 figures, and 16 tables.

Contents

S1. Supplementary methods 5
S1.1. Overarching study settings 5
S1.1.1. List of lignocellulose residues included in the study 5
S1.1.2. GLOBIOM model 6
S1.2. Regional availability of lignocellulose residues 9
S1.2.1. Residue-to-production ratios 9
S1.2.2. Theoretical potential of logging residues 10
S1.2.3. Summary of different potentials considered 11
S1.3. Global plastics production and embedded carbon 12
S1.4. Life-cycle inventories of lignocellulose residues 13
S1.4.1. Land use intensities and land use change 14
S1.4.2. Life-cycle inventories of agricultural residues 16
S1.4.3. Life-cycle inventories of forest residues 18
S1.4.4. Land use mapping 19
S1.5. Process simulation of biomass fractionation 19
S1.6. Life-cycle assessment of biobased plastics 24
S2. Supplementary results 26
S2.1. Lower-end estimation of the *available* potential 26
S2.2. Climate change impacts quantified with GTP100 27
S2.3. Impacts under the RCP1.9 scenario 28
S2.4. Impacts under the RCPref scenario 29
S2.5. Land use and its related impacts of lignocellulose residues 31
S2.6. Biomass sourcing strategies 35
S2.7. Contribution analysis of climate change impacts of biobased platform chemical 37
S3. Uncertainties and sensitivities 39
S3.1. Sensitivity analysis 39
S3.1.1. Residues price 39
S3.1.2. GLOBIOM model with endogenous supply of crop residues 39
S3.2. Uncertainties and limitations 43
S3.2.1. Product demand 43
S3.2.2. End-of-life biogenic CO_2 emissions 43
S3.3.3. Climate change impacts of intensified forest management 44
S3.3.4. Additional environmental impacts caused by lignocellulose residue removal 44
S3.3.5. Other uncertainties 45
S4. References 47

Table of Figures

Figure S1. Model structure....5
Figure S2. GLOBIOM regions....8
Figure S3. Carbon price and solid biomass demand depicted in GLOBIOM scenarios....8
Figure S4. Model structure for prospective LCA projects in Brightway2....13
Figure S5. Land use change allocation process for managed forest....15
Figure S6. Simplified process scheme of the biomass fractionation process....21
Figure S7. Global and regional available potentials of lignocellulose residues with the lower-end estimation....26
Figure S8. Projected climate change impacts of lignocellulose residues in 2050 quantified with the global temperature change potential over 100 years (GTP100)....27
Figure S9. Impact distributions of lignocellulose residues in 2050 under the RCP1.9 scenario across all nations....28
Figure S10. Projected climate change impacts of lignocellulose residues in 2050 under the RCPref scenario....29
Figure S11. Projected water stress and land-use-related biodiversity loss impacts of lignocellulose residues in 2050 under the RCPref scenario....30
Figure S12. Land use and its related impacts of lignocellulose residues from 2020 to 2050 in Brazil....31
Figure S13. Land use and its related impacts of lignocellulose residues from 2020 to 2050 in China....32
Figure S14. Land use and its related impacts of lignocellulose residues from 2020 to 2050 in India....33
Figure S15. Land use and its related impacts of lignocellulose residues from 2020 to 2050 in the USA....34
Figure S16. Impact trade-offs under the RCP1.9 scenario in 2050....35
Figure S17. Climate change, water stress, and biodiversity loss merit-order curves of lignocellulose residues in Brazil, China, India, and the United States under the RCP1.9 scenario in 2050....36
Figure S18. Climate change impacts of biobased platform chemicals based on propionaldehyde fractionation in 2050 under the RCP1.9 scenario....38
Figure S19. Sensitivity analysis: the impact of biomass residue price on climate change impacts of aggregated crop residues....41
Figure S20. Sensitivity analysis: solid biomass supply under RCP1.9....42
Figure S21. Sensitivity analysis: global land use under RCP1.9....43

Table of Tables

Table S1. List of lignocellulose residues included in the study 5
Table S2. List of GLOBIOM outputs......6
Table S3. Crop-specific empirical residue-to-product ratio (RPR) functions......9
Table S4. Constant RPRs. 10
Table S5. Different potentials considered for lignocellulose residues and key assumptions 11
Table S6. Global plastics production and embedded carbon in 2019 12
Table S7. List of updated inputs and emissions for LCIs of crop production......16
Table S8. List of regionalized ecoinvent activities for forest residues production 18
Table S9. Mapping between land-use classifications in GLOBIOM (LCI) and land-use related biodiversity loss life-cycle impact assessment (LCIA) method. 19
Table S10. Composition of the lignocellulose residues 22
Table S11. Bibliographic data used for the property estimation of the new Aspen components. .22
Table S12. Chemical reactions and conversions 23
Table S13. Design specifications for the solvent recovery distillation columns......23
Table S14. Life-cycle inventory of glucose produced from biomass fractionation. 24
Table S15. Life-cycle inventory of methanol produced from biomass gasification. 25
Table S16. Life-cycle inventory of propylene produced from methanol-to-olefin process......25

S1. Supplementary methods

S1.1. Overarching study settings

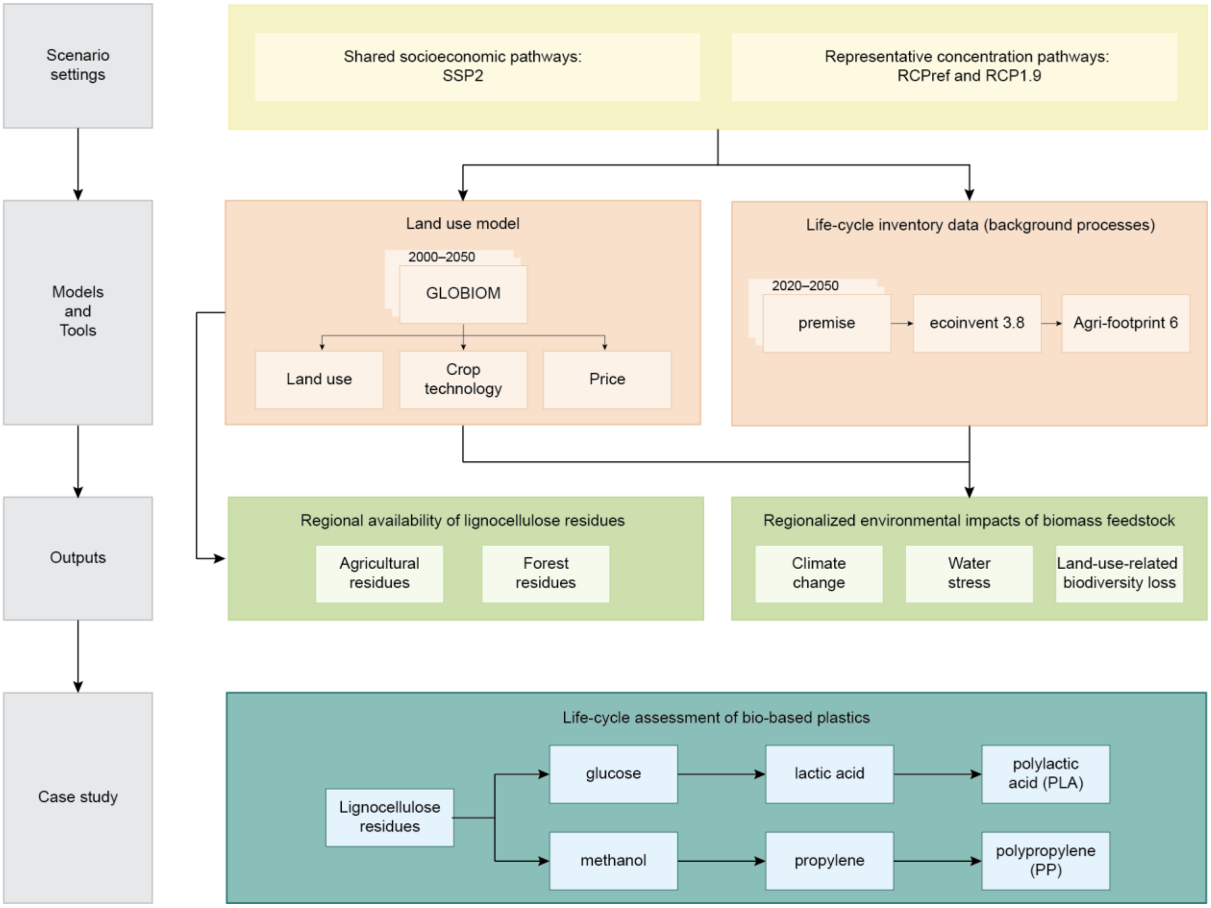

Figure S1. Model structure

S1.1.1. List of lignocellulose residues included in the study

Table S1. List of lignocellulose residues included in the study

	Harvest residues	Process residues
Agricultural residues	Barley straw<br>Maize straw<br>Rapeseed straw<br>Rice straw	Sugarcane bagasse*<br>Rice husks*

	Sorghum stray<br>Soybean straw<br>Sugarcane tops and leaves<br>Wheat straw	
Forest residues**	Logging residues, conifer wood<br>Logging residues, non-conifer wood	Sawdust, conifer wood<br>Sawdust, non-conifer wood<br>Wood chips, conifer wood<br>Wood chips, non-conifer wood

* Only included for availability study. LCA were not conducted due to data constraint

** Only from managed forests. Forest plantations are excluded from the study scope.

S1.1.2. GLOBIOM model

The Global Biosphere Management Model (GLOBIOM) is a partial equilibrium economic model that focuses on the agriculture, forest, and bioenergy sectors.[1, 2] It computes market equilibrium by optimizing total consumer and producer surpluses through the allocation of land use within these sectors.[1, 2] GLOBIOM is spatially explicit, operating at a spatial resolution of 200 km × 200 km for modeling land use and biomass production, while it models biomass demand, e.g. for food or as a building material, at the regional level.[3] Supplementary Table 2 presents a full list of the GLOBIOM outputs used in this study for the assessments of availability and associated environmental impact.

Table S2. List of GLOBIOM outputs

	**Spatial resolution**	**Used for**	**Unit**
GLOBIOM			
Cropland area by crop technology*	200 km × 200 km grid	LCA, land use-related inventory	1000 ha
Land use (unmanaged forest, managed forest, plantation, afforestation, cropland, grassland, other natural land, others)	200 km × 200 km grid	LCA, land use-related inventory	1000 ha
Price of agricultural and forest products	37 regions**	LCA, impact allocation	USD

Forest rotation period	179 countries	LCA, climate change impacts (sensitivity analysis)	Years
Sawnwood production<br>Pulpwood production	200 km × 200 km grid	Availability	1000 m^3
Stemwood yield<br>Sawlogs+pulpwood yield	200 km × 200 km grid	Availability, yield ratio of logging residue to pulpwood and sawnwood	m^3/ha
Sawnwood consumption for material<br>Pulpwood consumption for material<br>Sawnwood consumption for energy<br>Pulpwood consumption for energy	37 regions**	Availability, ratio of pulpwood and sawnwood consumption for material	1000 m^3
Sawdust production<br>Wood chips production	37 regions**	Availability	Mm^3
Crop production	200 km × 200 km grid	Availability	1000 t
Crop yield	200 km × 200 km grid	Availability, residue-to-crop ratio	t/ha
Nitrogen fertilizer inputs, by crop and crop technology<br>Phosphorous fertilizer inputs, by crop and crop technology	200 km × 200 km grid	LCA, fertilizer inventory	kg/ha
GLOBIOM-forest			
Sawnwood, conifer production<br>Sawnwood, non-conifer production<br>Pulpwood, conifer production<br>Pulpwood, non-conifer production	179 countries	Availability, ratio of conifer / non-conifer wood	1000 m^3
Forest land use (Primary forest, secondary forest, managed forest with three different intensities)	179 countries	LCA, land use-related inventory	Mha

* crop technology in GLOBIOM corresponds to different management intensity levels, see Table S9

** see Figure S2

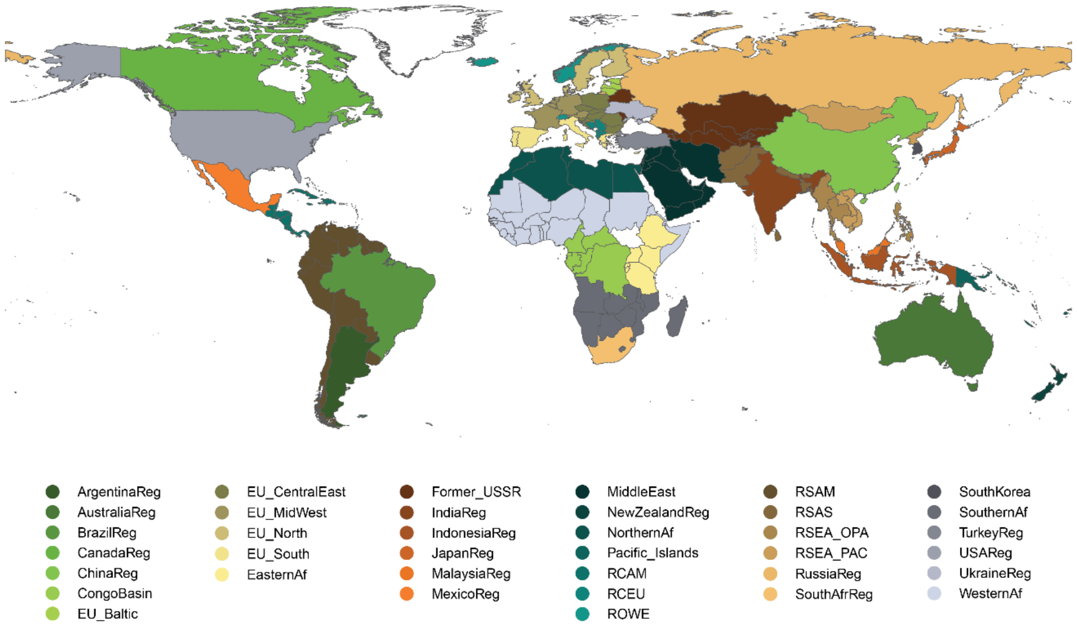

Figure S2. GLOBIOM regions

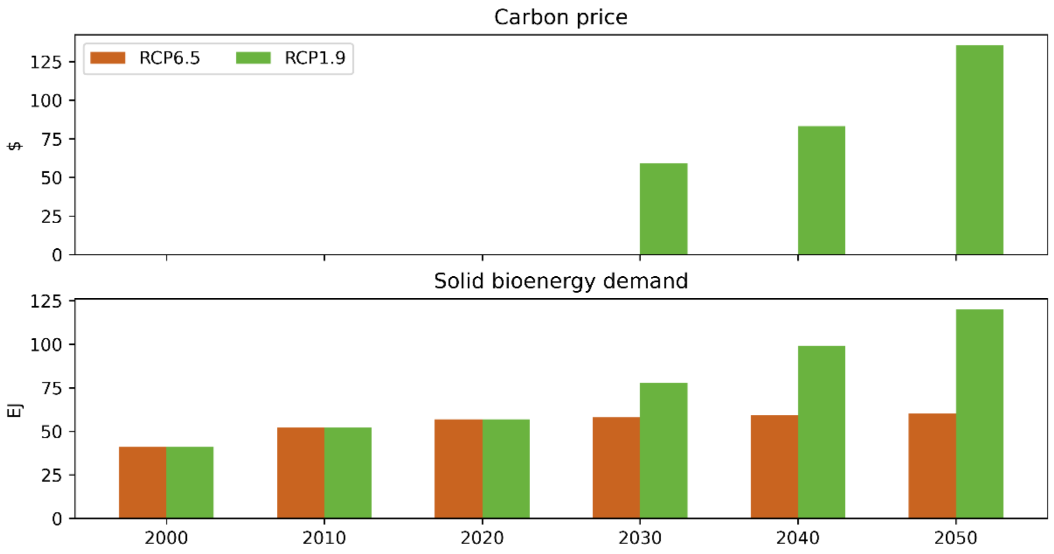

Figure S3. Carbon price and solid biomass demand depicted in GLOBIOM scenarios

* A carbon price in Integrated Assessment Models is a theoretical or modeled societal cost assigned to greenhouse gas emissions to simulate the economic impacts of carbon emissions and evaluate the effectiveness and costs of mitigation strategies.

S1.2. Regional availability of lignocellulose residues

S1.2.1. Residue-to-production ratios

The residue-to-production ratios were expressed as empirical functions of crop yields (y, in tonne dry mass/ha), which are spatially explicit outputs of the GLOBIOM model, and commonly take linear, logarithmic, or exponential forms (Table S3).[4] Constant RPRs (Table S4) are used to calculate process residue availability (rice husks and sugarcane bagasse) and harvest residues when empirical RPR functions are not available (Sugarcane tops and leaves).

Table S3. Crop-specific empirical residue-to-product ratio (RPR) functions.

**Form**	$\boldsymbol{RPR = a \times \exp(b \times y)}$ [4, 5]	$\boldsymbol{RPR = a \times \ln(b \times y)}$ [6]	$\boldsymbol{RPR = a \times y + b}$ [4]
Barley	a=1.822	a=-0.2751	a=-0.27
	b=-0.149	b=1.3796	b=2.77
Maize	a=2.656	a=-0.1807	a=-0.13
	b=-0.103	b=1.3373	b=2.20
Rice straw	a=2.450	a=-1.2256	a=-0.22
	b=-0.084	b=3.8450	b=2.56
Rapeseed	a=3.028	a=-0.4520	a=-0.60
	b=-0.200	b=2.0475	b=4.10
Soybeans	a=3.869	N.A.	a=-0.80
	b=-0.178		b=3.90
Sorghum	a=2.302	N.A.	a=-0.55
	b=-0.100		b=4.55
Wheat	a=2.183	a=-0.3629	a=-0.14
	b=-0.127	b=1.6057	b=1.96

Table S4. Constant RPRs.

	RPR **value**	**Reference**
Rice husks	0.20—0.36	[7, 8]
Sugarcane bagasse	0.23—0.37	[9]
Sugarcane tops and leaves	0.17—0.30	[9]

S1.2.2. Theoretical potential of logging residues

Key assumptions are listed below, in alignment with the GLOBIOM model[3, 10]:

- A tree is composed of 60% stemwood, 25% branches and stumps, and 15% of foliage and roots
- Foliage and roots are not harvested as logging residues
- Bark is 13.6% of a tree
- Wood density: $d_C = 0.45$ and $d_{NC} = 0.56$ tonne dry mass/m^3 for coniferous wood and non-coniferous wood, respectively[11]
- Logging residues are produced as a by-product of pulpwood and sawnwood for material use

The production of conifer and non-conifer logging residues ($M_{LR,C}$ and $M_{LR,NC}$, in 1000 tonne dry mass) are calculated on the grid level based on the production of pulpwood and sawnwood ($P_{PW} + P_{SW}$, reported in 1000 m^3, GLOBIOM model outputs on the grid level) according to Equation S1–S2,

$$M_{LR,C} = (P_{PW} + P_{SW}) \times r_{material} \times r_{LR\_to\_PW\_SW} \times r_C \times d_C \qquad \text{(S1)}$$

$$M_{LR,NC} = (P_{PW} + P_{SW}) \times r_{material} \times r_{LR\_to\_PW\_SW} \times (1 - r_C) \times d_{NC} \qquad \text{(S2)}$$

in which $r_{material}$ is the ratio of pulpwood and sawnwood consumption (GLOBIOM model outputs on the regional level, in 1000 m^3) for material.

$$r_{material} = \frac{C_{PW,material} + C_{SW,material}}{C_{PW,material} + C_{SW,material} + C_{PW,energy} + C_{SW,energy}} \qquad \text{(S3)}$$

$r_{LR\_to\_PW\_SW}$ is the yield ratio of logging residue to pulpwood and sawnwood, calculated from stemwood yield and sawnwood and pulpwood yield ($Y_{stemwood}$ and $Y_{sawnwood\_and\_pulpwood}$,

GLOBIOM model outputs on the grid level, in m^3/ha). 0.6 is the share of stemwood, 0.85 is the share of stemwood, branches and stumps, and 0.864 is the converting factor from under bark to over bark.

$$r_{LR\_to\_PW\_SW} = \frac{Y_{stemwood} \div 0.6 \times 0.85 - Y_{sawlogs\_and\_pulpwood} \div 0.864}{Y_{sawlogs\_and\_pulpwood} \div 0.864} \quad \text{S4)}$$

r_C is the ratio of conifer wood. Since the GLOBIOM model does not differentiate conifer and non-conifer wood, this information is based on the production of pulpwood and sawnwood from the GLOBIOM-forest model, a sub-model of GLOBIOM that provides a more comprehensive and detailed representation of the forest sector ($P'_{PW} + P'_{SW}$, GLOBIOM-forest model outputs on the country level, in 1000 m^3).

$$r_C = \frac{P'_{PW,C} + P'_{SW,C}}{P'_{PW,C} + P'_{SW,C} + P'_{PW,NC} + P'_{SW,NC}} \quad \text{(S5)}$$

S1.2.3. Summary of different potentials considered

Table S5. Different potentials considered for lignocellulose residues and key assumptions

**Residue types**		***Theoretical* potential (TP, [tonne DM/ha])**	***Ecological* potential (EP, [tonne DM/ha])**	***Available* potential (AP, [tonne DM/ha])**
Agricultural residues	Harvest residues	ex-post calculation based on main products	=Max(0, TP-2.5)	=0.7×EP
	Process residues	ex-post calculation based on main products	-	=0.7×TP
Forest residues	Harvest residues	ex-post calculation based on main products	-	=0.5×TP*
	Process residues	-	-	GLOBIOM model output

* included also ecological considerations

S1.3. Global plastics production and embedded carbon

Table S6. Global plastics production and embedded carbon in 2019

Plastics polymer	Global production in 2019 (Mt)[12]	Carbon content[13]	Embedded biogenic carbon (Mt)
PP	73	86%	63
Fibres	60	63%	38
HDPE	56	86%	48
LDPE, LLDPE	54	86%	47
PVC	51	38%	20
PET	25	63%	16
PS	21	92%	19
PUR	18	62%	11
ABS, ASA, SAN	9	85%	8
Elastomers (tyres)*	8	89%	7
Bioplastics**	2	73%	2
Road marking** coatings**	1	73%	1
Marine coatings**	1	73%	0
Other**	81	73%	59
**All**	**460**	**73%**	**338**

* Assuming carbon content of polybutadiene

** Global production-weighted average carbon content of other major plastics

Abbreviations: PP, polypropylene; HDPE, high-density polyethylene; LDPE: low-density polyethylene; LLDPE: linear low-density polyethylene; PVC, polyvinyl chloride; PET, Polyethylene terephthalate; PS, polystyrene; PUR, polyurethane; ABS, acrylonitrile-butadiene-styrene copolymer; ASA, acrylonitrile-styrene-acrylate copolymer; SAN, styrene-acrylonitrile copolymer

S1.4. Life-cycle inventories of lignocellulose residues

The environmental impacts associated with lignocellulose residues were assessed using prospective LCAs, implemented with the Brightway2 framework,[14] as outlined in Figure S4.

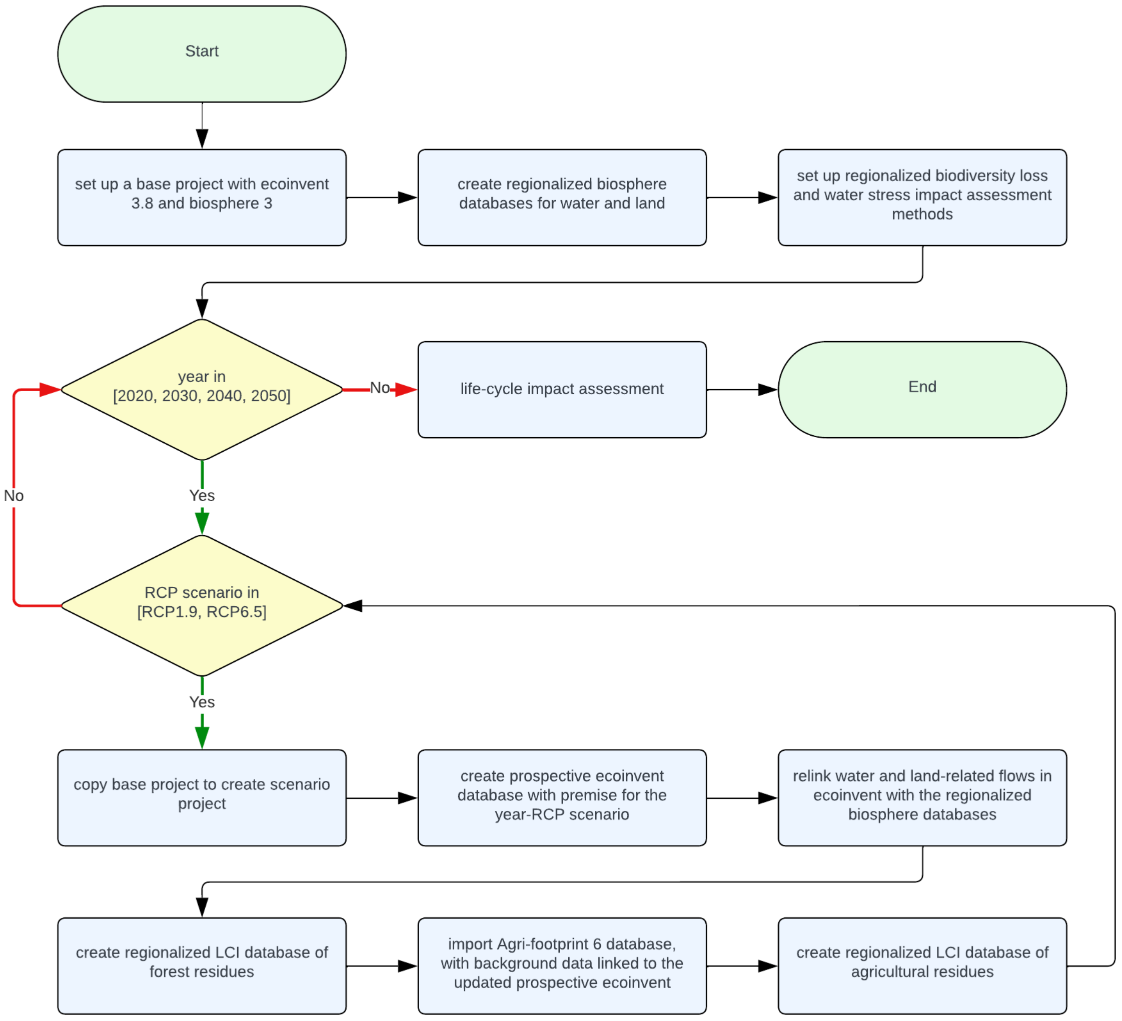

Figure S4. Model structure for prospective LCA projects in Brightway2

S1.4.1. Land use intensities and land use change

GLOBIOM considers possible land use changes between unmanaged forests, managed forests, afforestation, croplands, grasslands, and other natural lands. All other land-use types not involved in this land transition were grouped under the "others" category.

Differentiating the land-use intensities of croplands and managed forests enables a more precise impact assessment of land-use-related biodiversity loss. In GLOBIOM, the land-use intensity of croplands was differentiated with the endogenous parameter of crop management systems. Subsistence farming was associated with minimal land use, low-input rainfed systems with light land use, and high-input rainfed / irrigated systems with intense land use.

The assessment of different land-use intensities in the forest sector, on the other hand, was enabled through GLOBIOM-forest, a submodel of GLOBIOM that provides a more comprehensive and detailed representation of the forest sector. It encompasses three distinct forest types: primary forests, secondary forests, and managed forests. The "managed forests" category is further subdivided into three distinct management intensity levels—low intensity, multifunctional, and high intensity—thereby providing a detailed spectrum of forest management practices.[15] This level of detail enables a more comprehensive assessment of the land-use-related impacts of forestry products.

Allocation of the land-use changes for cropland was performed following the PAS 2050-1 Guidelines.[16] A similar allocation process was developed for managed forests (Figure S5). It is important to note that the expansion of secondary forests and afforestation are categorized independently and do not contribute to the impacts or credits associated with wood products harvested from managed forests.

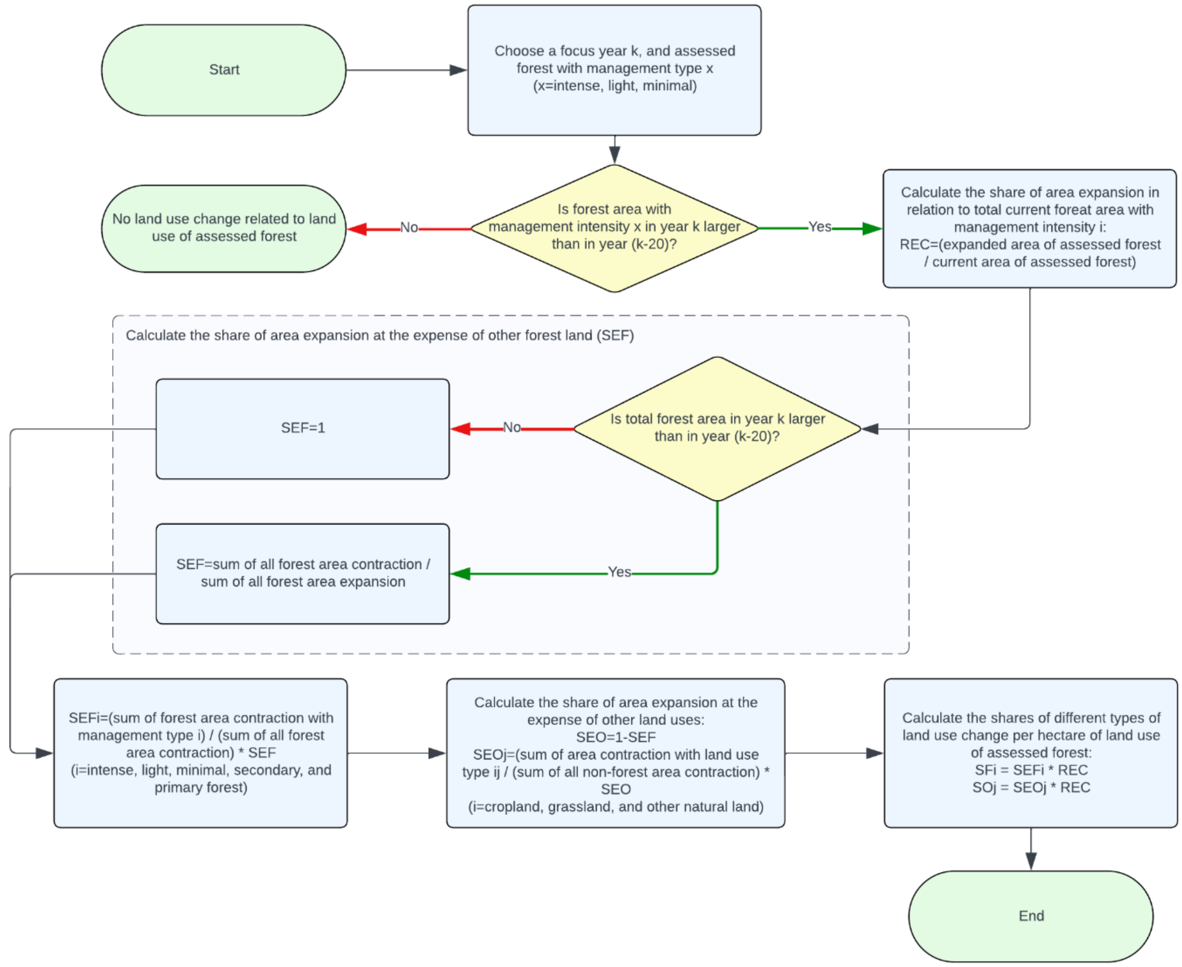

Figure S5. Land use change allocation process for managed forest

S1.4.2. Life-cycle inventories of agricultural residues

Table S7 provides a detailed description of the procedures used to create the inputs and emissions. Agri-footprint 6 incorporates ecoinvent 3.8 for its background processes such as fuel, electricity, and transportation. These processes were relinked with regionalized and prospective premise-generated background LCIs. In this study, we extended our analysis with more countries for each crop than is covered by Agri-footprint 6. For the inputs and emissions not specifically modeled in our study, we calculated a global average for each crop. We did this by taking the production amount from countries included in Agri-footprint 6 and using them to weigh these averages. We then merged these global averages with the region-specific data we modeled, applying them to the countries that fell outside the scope of Agri-footprint 6. These calculated values were directly applied to all the countries that were not included. The impacts from the reference flow of one hectare of cropland were allocated to crops and their corresponding *ecological* potential of harvest residues, according to their economic values. However, as agricultural residues were not included in the GLOBIOM model as products, it was assumed that per kilogram agricultural residues shared the same price as forest residues. The impacts of agricultural process residues (rice husks and sugarcane bagasse) were not modeled.

Table S7. List of updated inputs and emissions for LCIs of crop production

(reference flow: one hectare of cropland)

**Inputs and emissions**	**Description**
Land occupation	1 ha of cropland, as the reference flow. The share of each cropland use intensity was categorized for country-crop combinations based on the share of harvest area with the corresponding crop technology, with details in Table S9.
Land transformation	Following PAS 2050-1 guideline,[16] the area of each land use type was assessed for the reference year and 20 years before that. Land use change was assumed to distribute equally over the 20 years. The model structure for the detailed calculation steps was updated based on PAS 2050-1 guideline by taking primary forest into account.
Blue water consumption	Pfister *et al.*[17] with 832 country-crop combinations for the considered crop types. The reported blue water consumption is in the unit of m^3 water/tonne crop. It was multiplied by the crop yield (tonne crop/ha) to match the reference flow.
Fertilizer	Nitrogen and phosphorus fertilizer application rate [kg/ha] is embedded in the crop technology parameter from the GLOBIOM model at the 200 km × 200 km resolution gird level for each crop. As potassium fertilizer is not included in GLOBIOM model, the data from the fertilizer use

	by crop (FUBC) survey data published by the International Fertilizer Association was used instead[18]. Since there is future projections in FUBC, it was assumed that the application rate of potassium fertilizer would increase with the same ratio as nitrogen fertilizer in future scenarios following Eq. S6.<br><br>$$Fer_{i,j,k}^{K} = \frac{Fer_{i,j,k}^{N-GLOBIOM}}{Fer_{i,j,2020}^{N-FUBC}} \times Fer_{i,j,2020}^{K-FUBC} \quad (S6)$$<br><br>$Fer_{i,j,k}^{K}$ [kg/ha]: derived potassium fertilizer application rate for crop i in country j in year k.<br><br>$Fer_{i,j,2020}^{N-FUBC}$ [kg/ha]: potassium fertilizer application rate for crop i in country j reported by FUBC.<br><br>$Fer_{i,j,k}^{N-GLOBIOM}$ [kg/ha]: nitrogen fertilizer application rate for crop i in country j in year k according to the GLOBIOM model.<br><br>$Fer_{i,j,2020}^{N-FUBC}$ [kg/ha]: nitrogen fertilizer application rate for crop i in country j reported by FUBC.<br><br>To avoid unreasonably high application, the highest possible application rate of potassium fertilizer was assumed to be the 95th percentile of the application rate in the FUBC database.<br><br>To match the aggregated NPK fertilizer application with each fertilizer product, the average agricultural use of each fertilizer product from 2018 to 2020 on the country level from the Food and Agriculture Organization of the United Nations (FAO) database was used.[19] The matching procedure followed Agri-footprint 6 methodology report.[20]
Land use change emissions	The considered GHG emissions from land use change include CO_2 emissions from net carbon change in biomass, and net soil organic carbon (SOC) change in mineral soils. The calculation steps followed Agri-footprint 6 methodology report[20] and the IPCC guideline,[21] which below specified data sources:<br><br>The above ground and below ground carbon stock in forest by country reported in Global Forest Resources Assessments by FAO[22] was used in this study. The default of carbon stock in annual and perennial croplands (4 and 20 tonne C/ha respectively) were used according to PAS 2050-1 guideline[16]. The carbon stock in grassland varies according to climate and soil types, with default values in each category from the IPCC guideline.[21] The climate zone maps[21] and the harmonized world soil database v1.2[23] were used to derive the share of each climate-soil combination on the country level.
Fertilizer emissions	The updated field emissions as a result of fertilizer application include CO_2 from urea, direct and indirect N_2O, NH_3 and NO_3^-–N emissions from nitrogen fertilizers. IPCC tier 1 emission factors and constants[24] were used in the calculation.

Crop residue emissions	Due to a lack of data on the fraction of crop residues being incinerated on the field for future scenarios, it was assumed that the entire unharvested crop residue is left in the field, and thus lead to direct and indirect N_2O emissions. IPCC tier 1 emission factors and constants[24] were used in the calculation.

S1.4.3. Life-cycle inventories of forest residues

Table S8. List of regionalized ecoinvent activities for forest residues production

**Machinery and other background activities: regionalized on the IMAGE-region level**
Cable yarding | yarding and processing, mobile cable yarder on truck
Cable yarding | yarding, mobile cable yarder on trailer
Cable yarding | yarding, sled yarder
Clefting of energy wood | clefting/splitting of energy wood
Delimbing/sorting, excavator-based processor | delimbing, with excavator-based processor
Forwarding, forwarder | forwarding, forwarder
Gravel, crushed | market for gravel, crushed
Harvesting, forestry harvester | harvesting, forestry harvester
Skidding, skidder | skidding, skidder
Diesel, burned in building machine
**Harvesting activities: regionalized on the country level**
Cleft timber, measured as dry mass | hardwood forestry, mixed species, sustainable forest management **(logging residue, hardwood)**
Cleft timber, measured as dry mass | softwood forestry, mixed species, sustainable forest management **(logging residue, softwood)**
Sawlog and veneer log, hardwood, measured as solid wood under bark | hardwood forestry, mixed species, sustainable forest management
Sawlog and veneer log, softwood, measured as solid wood under bark | softwood forestry, mixed species, sustainable forest management
**Sawmill activities: regionalized on the country level**
Sawnwood, hardwood, raw | sawing, hardwood

Slab and siding, hardwood, wet, measured as dry mass | sawing, hardwood **(wood chips, hardwood)**	
Sawdust, loose, wet, measured as dry mass | sawing, hardwood **(sawdust, hardwood)**	
Sawnwood, softwood, raw | sawing, softwood	
Slab and siding, softwood, wet, measured as dry mass | sawing, softwood **(wood chips, softwood)**	
Sawdust, loose, wet, measured as dry mass | sawing, softwood **(sawdust, softwood)**	

* Considered forest residues in this study are marked bold.

S1.4.4. Land use mapping

The mapping between the background LCI (ecoinvent 3.8) and the LCIA method follows Scherer *et al.*[25]

Table S9. Mapping between land-use classifications in GLOBIOM (LCI) and land-use related biodiversity loss life-cycle impact assessment (LCIA) method.

GLOBIOM	LCIA
Primary forest	Not assigned, natural state
Secondary forest	Managed forest, minimal
Managed forest, low intensity	Managed forest, minimal
Managed forest, multifunctional	Managed forest, light
Managed forest, high intensity	Managed forest, intense
Cropland, subsistence farming	Cropland, minimal
Cropland, low input rainfed	Cropland, light
Cropland, high input rainfed	Cropland, intense
Cropland, high input irrigated	Cropland, intense
Grassland	Grassland, intense
Other natural land	Not assigned, natural state

S1.5. Process simulation of biomass fractionation

The process simulation of the biomass fractionation process was developed in Aspen Plus v12 and it is based on the work of Talebi Amiri *et al*.[26] More specifically, on steps 35 to 83 of the propionaldehyde biomass fractionation protocol to afford cellulose, stabilized lignin and dipropylxylose (DPX) (Figure S6). We assumed an initial biomass composition of birch wood

extracted from the same work, excluding the minor sugars, acid-soluble lignin and acetyl (Table S10).

The components xylan, arabinan, lignin, stabilized lignin, glucan and DPX were created as new Aspen components. We estimated the missing property data of the new components with the UNIFAC method built in Aspen Plus v12.

Table S11 collects the initial data used for the component property estimation.

The process simulation mass flows were adapted from the original laboratory protocol to reflect a potential industrial-scale capacity. For example, the initial components entering the process (biomass, propionaldehyde, 1,4-dioxane and 37% wt. hydrochloric acid) were linearly scaled from 4.5 g, 4.8 mL, 25 mL and 0.85 mL to 45 kg/h, 48 L/h, 25 L/h and 8.5 L/h, respectively. Operating temperatures and pressures were faithfully adapted from the protocol.

Due to the lack of specific data about the separations and the solid/solvent interactions, extractions and washes were assumed to behave ideally (i.e., the totality of the desired fraction to be removed with the solvent, and the solvent itself, is successfully recovered). Additionally, on top of the already existing vacuum flash drums described in the original protocol, several distillation columns were added to the process to recover and recycle most of the solvents used in these extraction and wash steps. Table S13 shows the specifications of these columns. Furthermore, a purge of 0.1% of each recycle stream was assumed to consider potential losses.

Due to the lack of a vacuum pump unit in Aspen Plus, electricity consumption associated with vacuum generation was approximated to the work required to recompress the subatmospheric pressure streams after the flash drum separation back to 1 bar.

The main reactor is modeled as an isothermal conversion reactor, while the neutralization reactors and the cellulose hydrolysis reactor are considered adiabatic. The chemical reactions taking place and their respective conversion are displayed in Table S13.

In the furnace, all unseparated solvents and unretrieved biomass are combusted using air and considering 100% conversion. The energy of the process cold and hot streams is integrated considering the targets provided by Aspen Energy Analyzer.

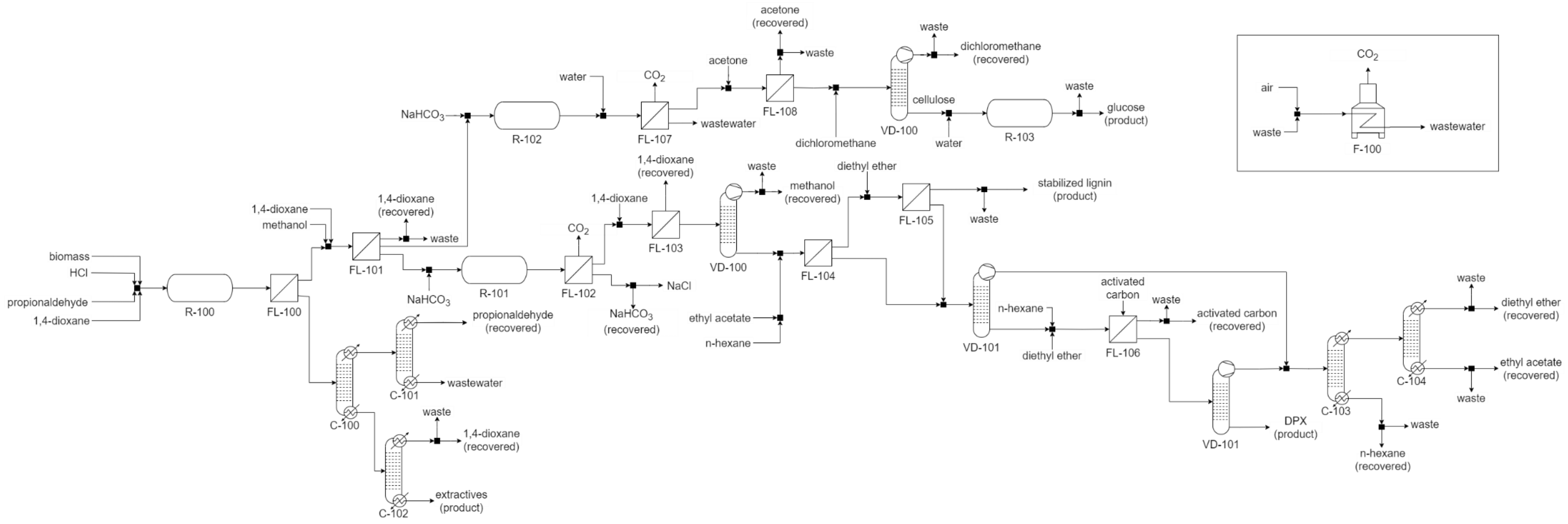

Figure S6. Simplified process scheme of the biomass fractionation process.

R symbolizes chemical reactors; D, distillation columns; VD, vacuum flash drums; FL, filters and F, furnaces.

Table S10. Composition of the lignocellulose residues

Component	Mass fraction (Talebi Amiri et al.[26])	Mass fraction (this work)
Xylan	0.1781	0.2224
Arabinan	0.0281	0.0351
Glucan	0.3216	0.4016
Lignin	0.1794	0.2240
Extractives	0.0327	0.0408
Water	0.0609	0.0760
Acid-soluble lignin	0.0281	-
Minor sugar	0.0283	-
Acetyl	0.0589	-

Table S11. Bibliographic data used for the property estimation of the new Aspen components.

Component	Formula	Solid standard enthalpy of formation [kJ/mol]	Solid molar density [cm^3/kmol]
Xylan[27]	$C_5H_8O_4$	$-7.624 \cdot 10^2$	86.40
Arabinan[27]	$C_5H_8O_4$	$-7.624 \cdot 10^2$	86.40
Lignin[26, 27]	$C_{103}H_{124}O_{40}$	$-1.593 \cdot 10^4$	79.50
Stabilized lignin[26, 27]	$C_{106}H_{128}O_{40}$	$-1.593 \cdot 10^4$	79.50
Glucan[27]	$C_6H_{10}O_5$	$-9.764 \cdot 10^2$	106.00
DPX[26]	$C_{11}H_{18}O_5$	-	-

Extractives were approximated to luteoline ($C_{15}H_{10}O_6$), whose properties are already present in the Aspen database.

Table S12. Chemical reactions and conversions

of the main (1 – 4), neutralization (5) and cellulose hydrolisys (6) reactors.

**Reaction**		**Conversion [%]**
1	$C_5H_8O_4$ (xylan) + H_2O → $C_5H_{10}O_5$ (xylose)	100
2	$C_5H_{10}O_5$ (xylose) + 2 C_3H_6O (propionaldehyde) → $C_{11}H_{18}O_5$(DPX) + 2 H_2O	60
3	$C_{103}H_{124}O_{40}$(lignin) + C_3H_6O (propionaldehyde) → $C_{106}H_{128}O_{40}$ (stabilized lignin) + H_2O	100
4	$C_5H_8O_4$ (arabinan) + H_2O → $C_5H_{10}O_5$ (arabinose)	100
5	HCl + NaHCO3 → NaCl + H_2O + CO_2	100
6	$C_6H_{10}O_5$ (cellulose) + H_2O → $C_6H_{12}O_6$ (glucose)	100

Table S13. Design specifications for the solvent recovery distillation columns.

All columns work at atmospheric conditions with a total condenser. The reflux ratio is taken as 1.3 times the minimum reflux ratio.

**Column**	**Light key**	**Recovery**	**Heavy key**	**Recovery**
C-100	Propionaldehyde	0.9999	1,4-dioxane	0.0001
C-101	Propionaldehyde	0.9999	Water	0.0001
C-102	1,4-dioxane	0.9999	Extractives	0.0001
C-103	Ethyl acetate	0.9999	N-hexane	0.0001
C-104	Diethyl ether	0.9999	Ethyl acetate	0.0001

S1.6. Life-cycle assessment of biobased plastics

Table S14 to Table S16 are life-cycle inventories of glucose, methanol, and propylene used for the biobased plastics. The life-cycle inventories of lactic acid, polypropylene and polylactic acid are based on the Process Economics Program Yearbook[28] that require an additional license from IHS Markit.

Table S14. Life-cycle inventory of glucose produced from biomass fractionation.

**Flow name**	**Amount**	**Unit**
Output		
Glucose	1	kg
Input		
Lignocellulose residue	1.00E+00	kg
Dioxane	1.03E-02	kg
HCl 30%	8.13E-04	kg
Propionaldehyde	1.23E-01	kg
Methanol	1.51E-02	kg
Bicarbonate	1.41E-03	kg
Water	1.46E+01	kg
Acetone	3.50E-03	kg
Dichloromethane	3.67E-02	kg
Ethylacetate	3.47E-04	kg
Hexanes	4.48E-02	kg
Diethyl ether	1.12E-04	kg
Activated carbon	2.22E-04	kg
Air	2.22E+00	kg
Cooling	5.30E+01	MJ
Electricity	4.91E+00	kWh
Emissions to air		
CO_2 (fossil)	3.14E-01	kg
CO_2 (biogenic)	5.18E-01	kg
Hydrochloric acid	3.14E-02	kg
Emissions to water		
Water	3.01E-01	kg
Hydrochloric acid	9.26E-05	kg
Propionaldehyde	7.20E-05	kg

Table S15. Life-cycle inventory of methanol produced from biomass gasification.

**Flow name**	**Amount**	**Unit**
Output		
Methanol	1	kg
Input		
Lignocellulose residue	2.60*	kg
Emissions to air		
CO_2 (biogenic)	3.39**	kg

* including both feedstock and fuel use.[29]

** assuming all carbon from the lignocellulose residue that is not embedded in the methanol is released as biogenic CO_2. It includes fuel emissions as well as process emissions.

Table S16. Life-cycle inventory of propylene produced from methanol-to-olefin process

The inventory data is based on Hoppe *et al.*[30]

**Flow name**	**Amount**	**Unit**
Output		
Propylene	1	kg
Input		
Methanol	2.57	kg
Electricity	0.46	kWh
Steam	5.59	MJ

S2. Supplementary results

S2.1. Lower-end estimation of the *available* potential

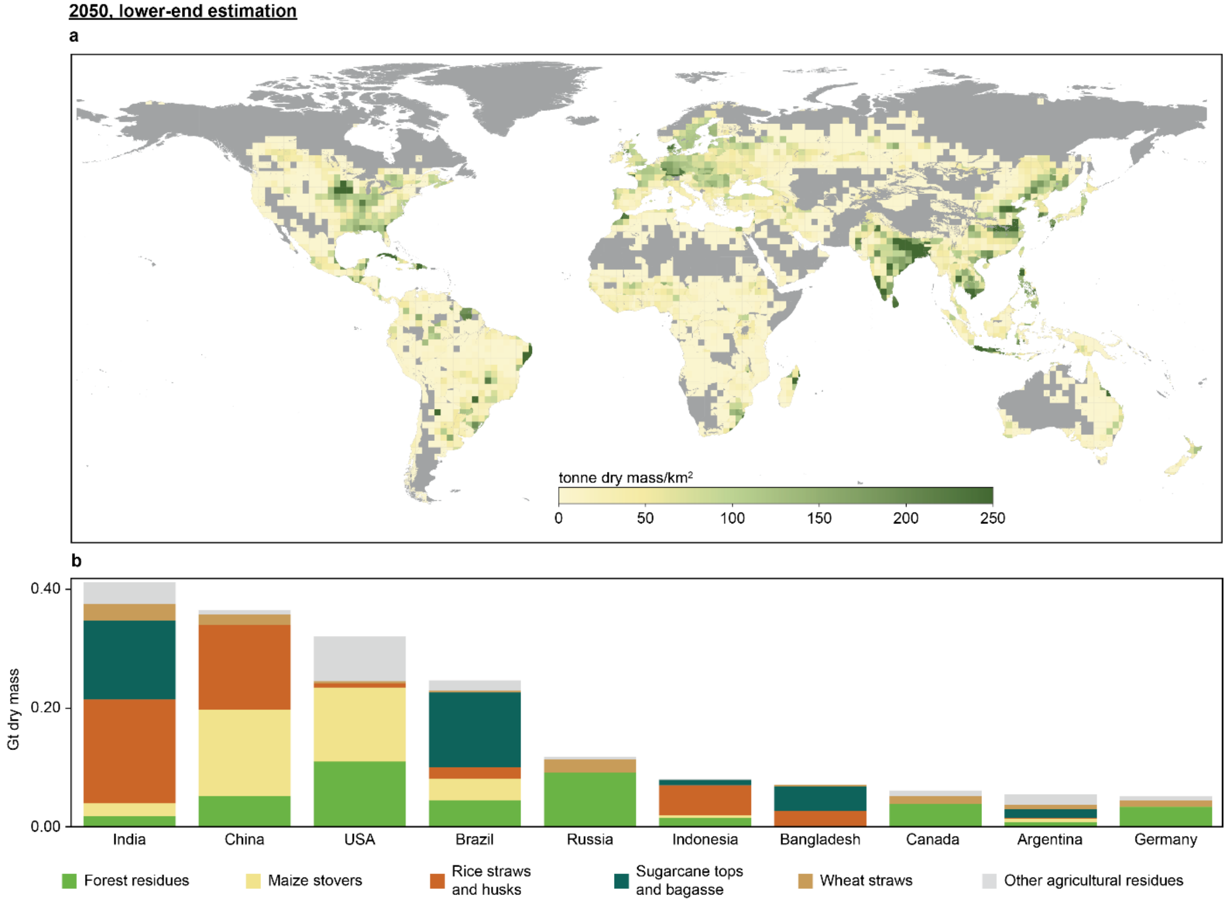

Figure S7. Global and regional available potentials of lignocellulose residues with the lower-end estimation.

a) Spatial distribution of lignocellulose residues in 2050 in 200 km × 200 km resolution. The gray area reflects no cropland or managed forest in the specific region. b) Top 10 countries with the highest potential for lignocellulose residues by biomass type in 2050.

S2.2. Climate change impacts quantified with GTP100

GTP 100a

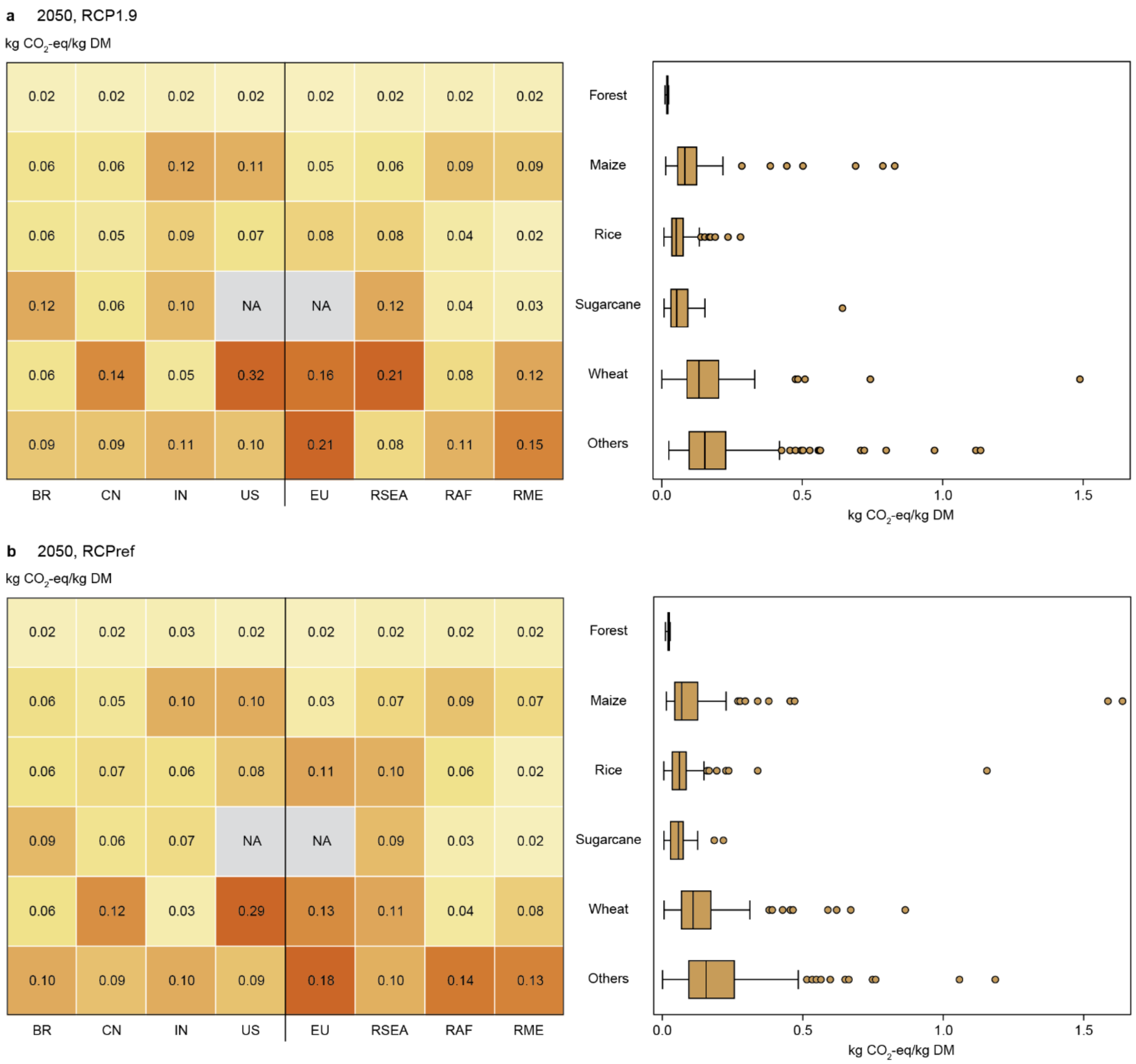

Figure S8. Projected climate change impacts of lignocellulose residues in 2050 quantified with the global temperature change potential over 100 years (GTP100).

a) under the RCP1.9 scenario; b) under the RCPref scenario. The impacts of agricultural residues are quantified with only harvest residues. The impacts of forest residues are based on the availability-weighted average of both harvest and process residues. Region abbreviations: BR, Brazil; CN, China; IN, India; US: the United States of America; EU, the European Union; RSEA: Region South East Asia; RAF, Region Africa; RME, Region Middle East.

S2.3. Impacts under the RCP1.9 scenario

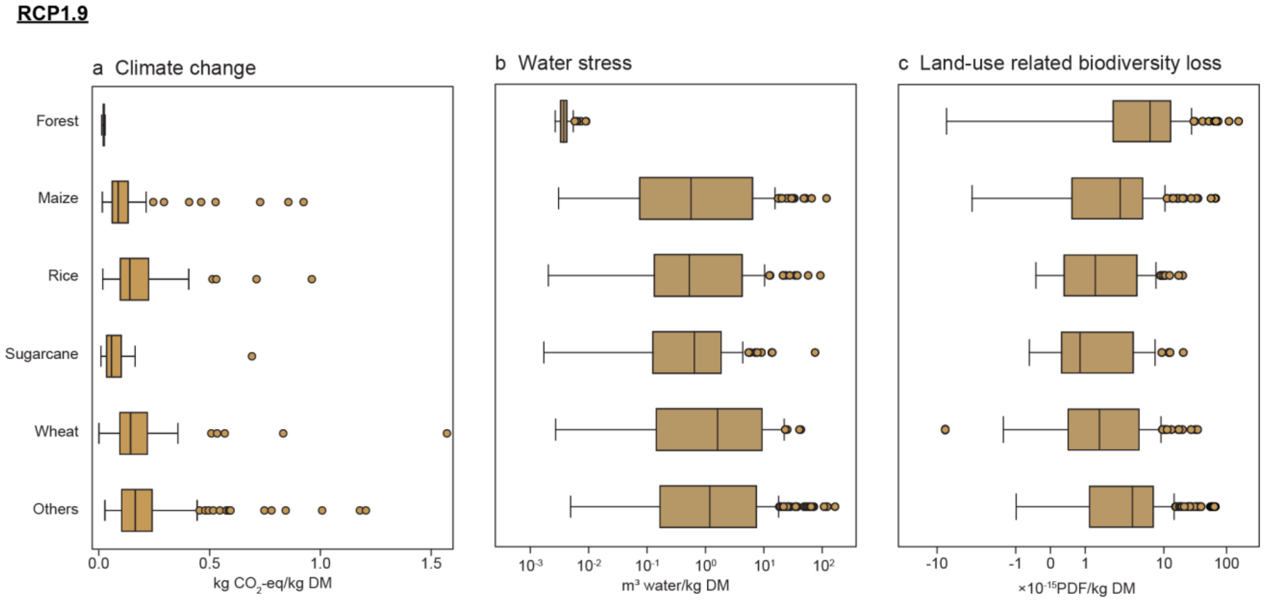

Figure S9. Impact distributions of lignocellulose residues in 2050 under the RCP1.9 scenario across all nations.

The impacts of agricultural residues are quantified with only harvest residues. The impacts of forest residues are based on the availability-weighted average of both harvest and process residues.

S2.4. Impacts under the RCPref scenario

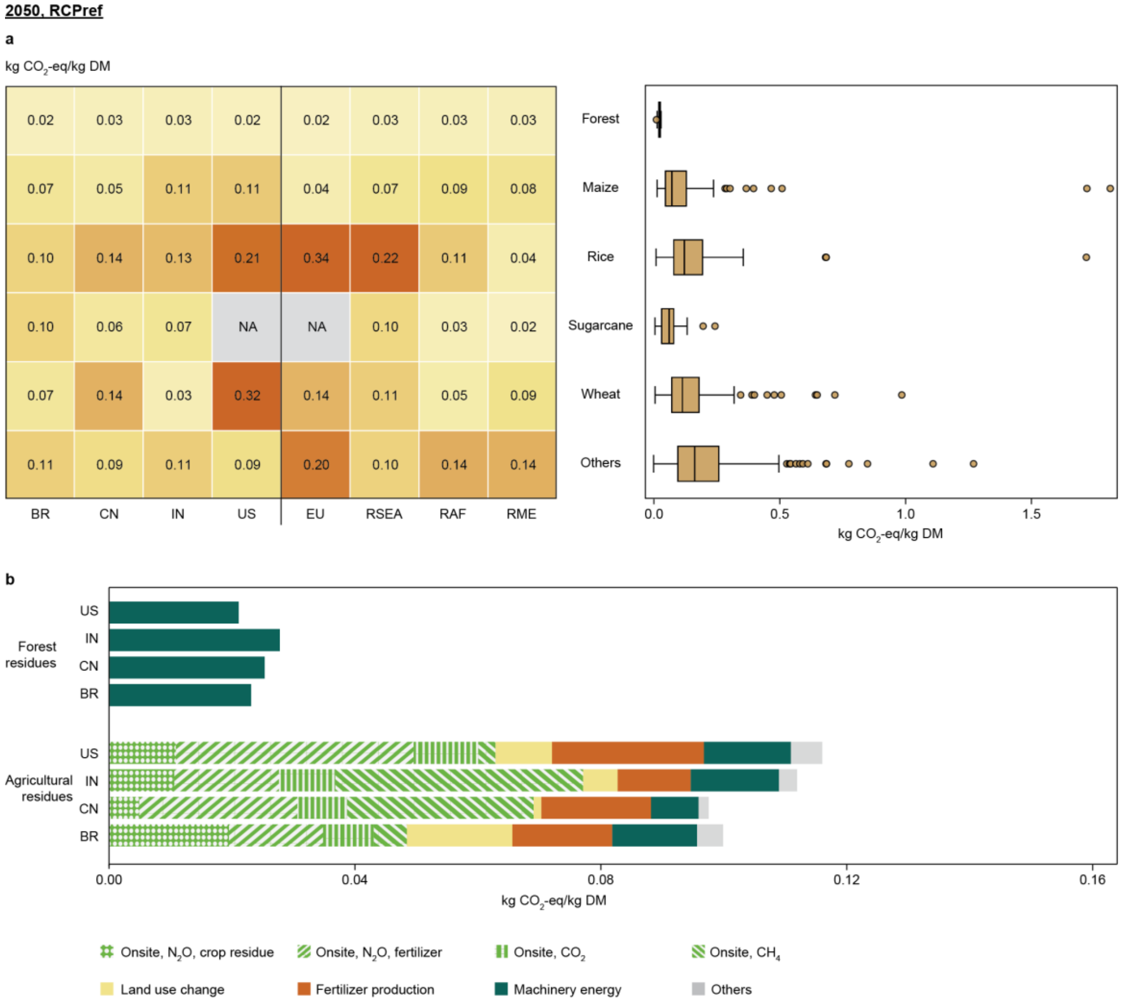

Figure S10. Projected climate change impacts of lignocellulose residues in 2050 under the RCPref scenario.

a) Heatmap of the cradle-to-gate climate change impacts quantified as the global warming potential over 100 years (GWP100) associated with each biomass type in selected countries and regions (left), and the distributions across all countries (right). b) Cradle-to-gate contribution analysis of the climate change impacts (GWP100) associated with forest and agricultural residues in the four countries with the highest lignocellulose residue potential. The impacts of agricultural residues are quantified with only harvest residues. The impacts of forest residues are based on the availability-weighted average of both harvest and process residues. Abbreviations: DM, dry mass; BR, Brazil; CN, China; IN, India; US: the United States of America; EU, the European Union; RSEA: Region South East Asia; RAF, Region Africa; RME, Region Middle East.

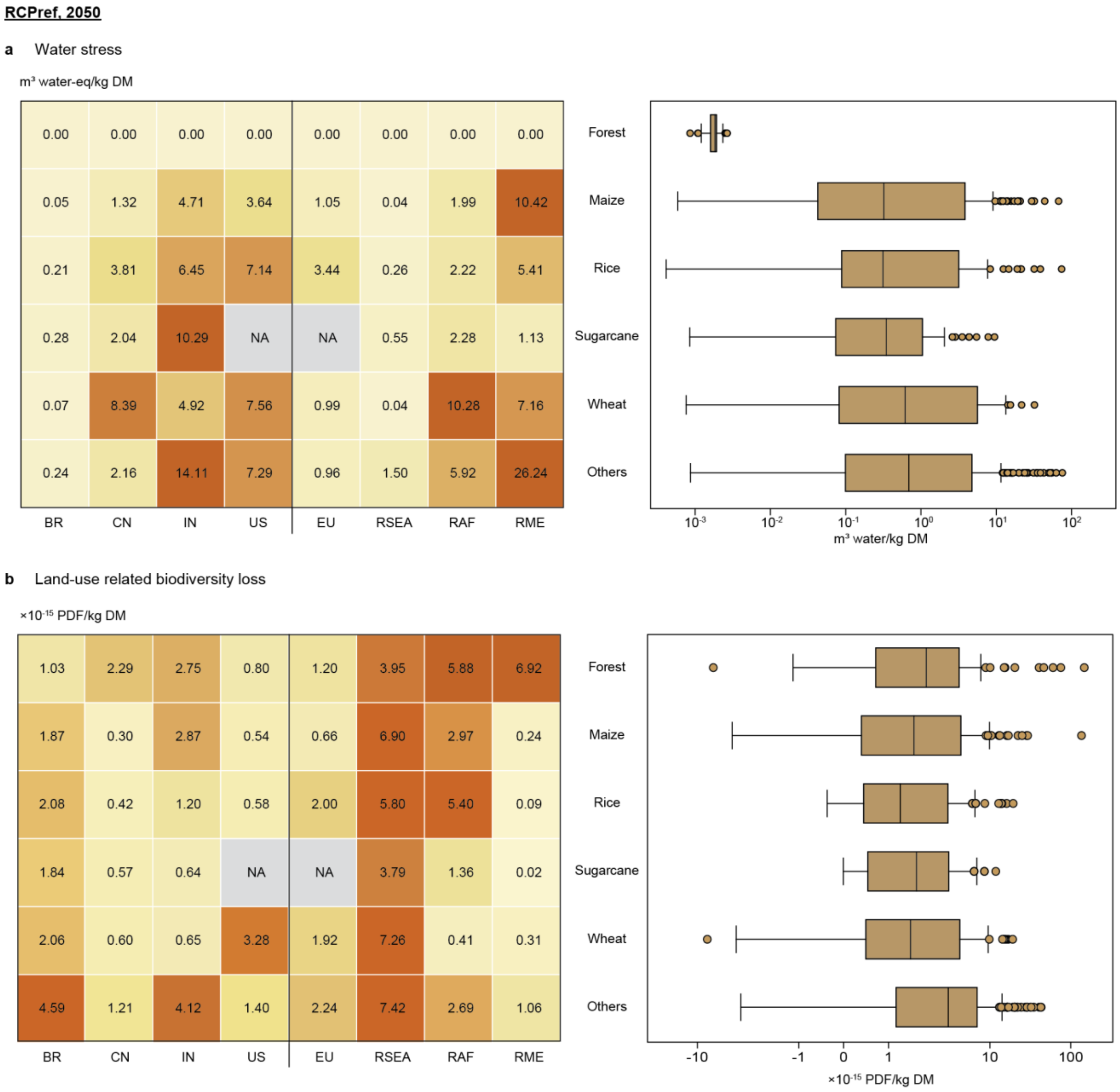

Figure S11. Projected water stress and land-use-related biodiversity loss impacts of lignocellulose residues in 2050 under the RCPref scenario.

a) and b) Heatmaps of the cradle-to-gate water stress and land-use-related biodiversity loss, respectively, associated with each biomass type in selected countries and regions (left) and the distribution across all countries (right). The impacts of agricultural residues are quantified with only harvest residues. The impacts of forest residues are based on the availability-weighted average of both harvest and process residues. Abbreviations: PDF: potentially disappeared fractions of species; DM, dry mass; BR, Brazil; CN, China; IN, India; US: the United States of America; EU, the European Union; RSEA: Region South East Asia; RAF, Region Africa; RME, Region Middle East.

S2.5. Land use and its related impacts of lignocellulose residues

Figure S12 – Figure S15 represent land use and its related impacts of lignocellulose residues from 2020 to 2050 in Brazil, China, India and the USA respectively.

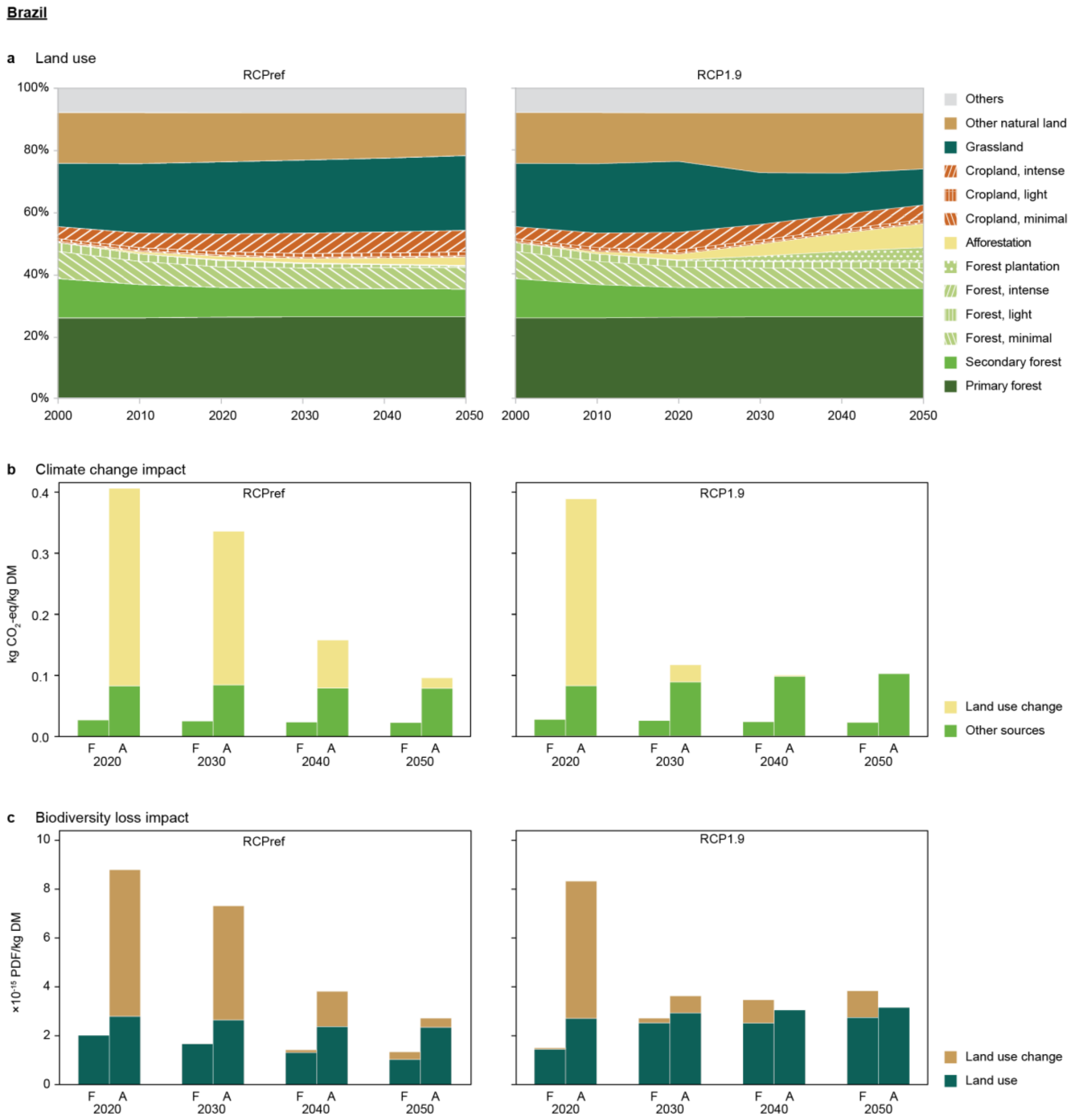

Figure S12. Land use and its related impacts of lignocellulose residues from 2020 to 2050 in Brazil.

a) Land-use percentages under RCPref and RCP1.9. b) and c) Climate change impacts and land-use-related biodiversity loss impacts, respectively, of forest residues and agricultural residues contributed by land-use change and others factors under RCPref and RCP1.9. Other main sources contributing to the climate change impacts include onsite emissions, fertilizer production and machinery energy. Abbreviations: F, forest residues; A, agricultural residues.

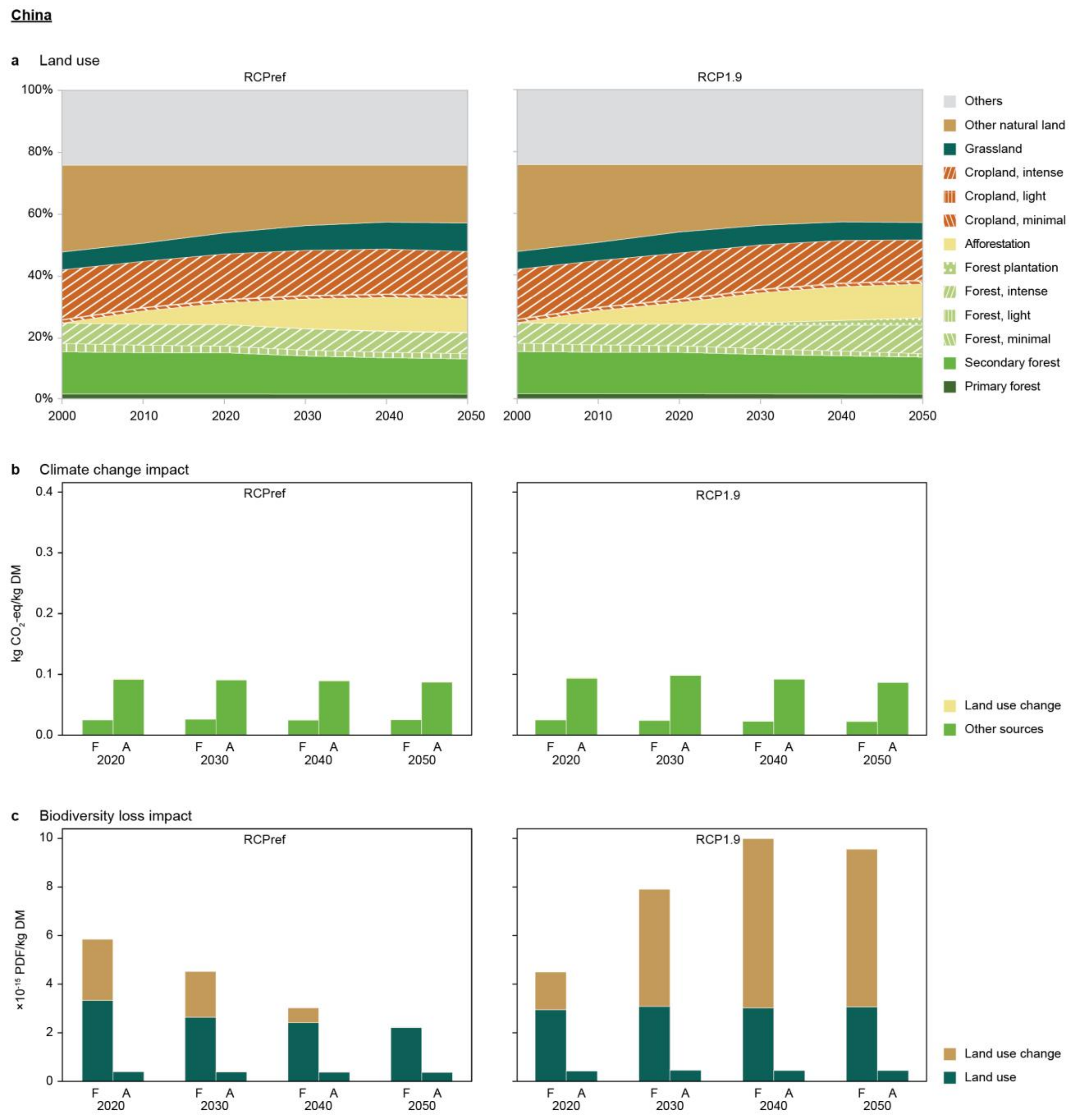

Figure S13. Land use and its related impacts of lignocellulose residues from 2020 to 2050 in China.

a) Land-use percentages under RCPref and RCP1.9. b) and c) Climate change impacts and land-use-related biodiversity loss impacts, respectively, of forest residues and agricultural residues contributed by land-use change and others factors under RCPref and RCP1.9. Other main sources contributing to the climate change impacts include onsite emissions, fertilizer production and machinery energy. Abbreviations: F, forest residues; A, agricultural residues.

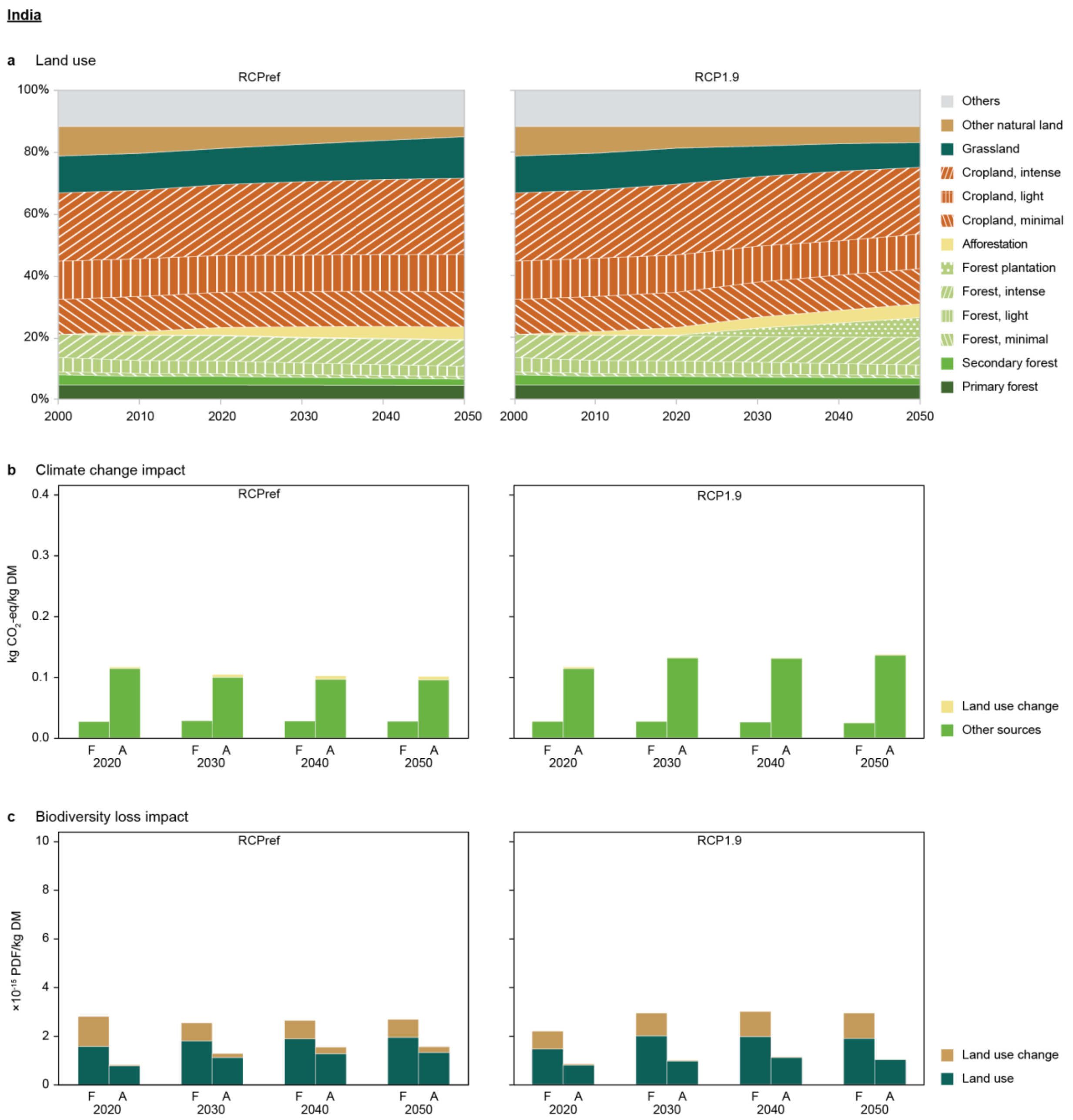

Figure S14. Land use and its related impacts of lignocellulose residues from 2020 to 2050 in India.

a) Land-use percentages under RCPref and RCP1.9. b) and c) Climate change impacts and land-use-related biodiversity loss impacts, respectively, of forest residues and agricultural residues contributed by land-use change and others factors under RCPref and RCP1.9. Other main sources contributing to the climate change impacts include onsite emissions, fertilizer production and machinery energy. Abbreviations: F, forest residues; A, agricultural residues.

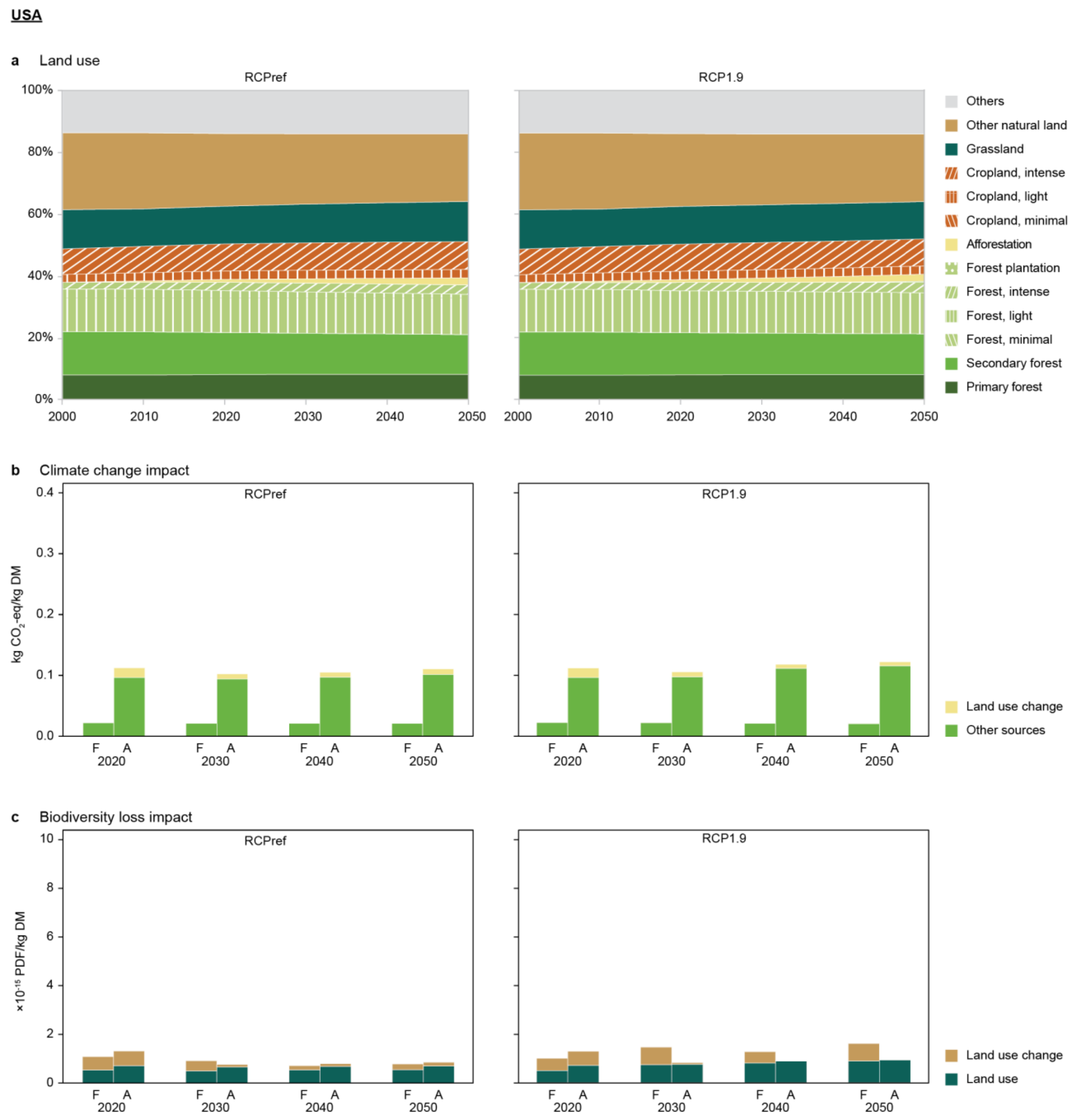

Figure S15. Land use and its related impacts of lignocellulose residues from 2020 to 2050 in the USA.

a) Land-use percentages under RCPref and RCP1.9. b) and c) Climate change impacts and land-use-related biodiversity loss impacts, respectively, of forest residues and agricultural residues contributed by land-use change and others factors under RCPref and RCP1.9. Other main sources contributing to the climate change impacts include onsite emissions, fertilizer production and machinery energy. Abbreviations: F, forest residues; A, agricultural residues.

S2.6. Biomass sourcing strategies

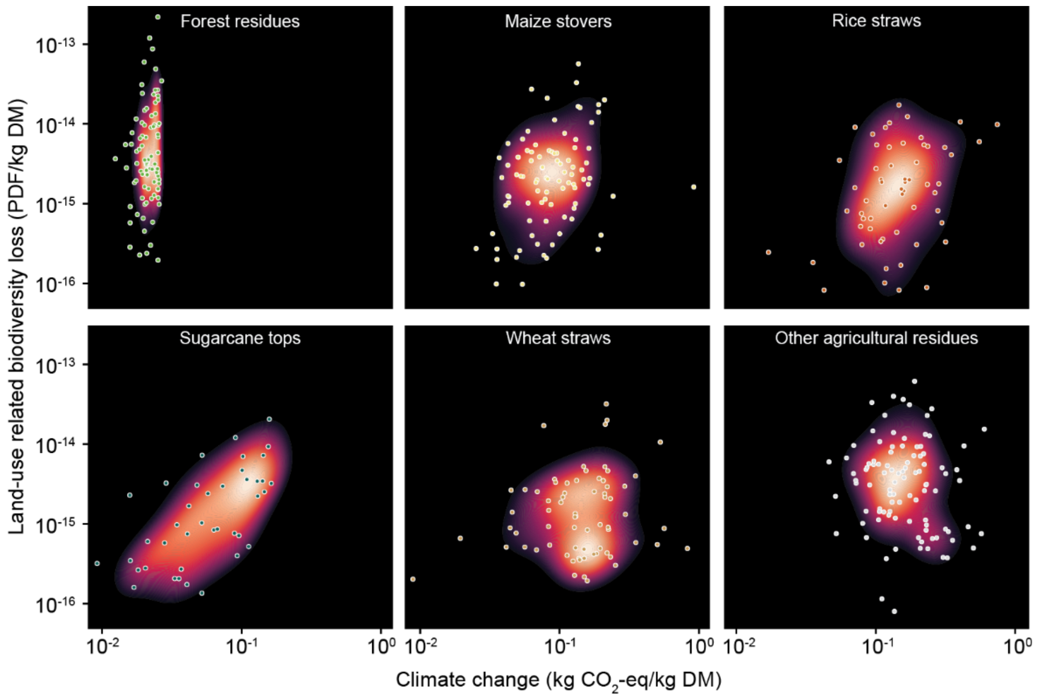

*Figure S16. Impact trade-offs under the RCP1.9 scenario in 2050***.**

Kernel density estimations of biodiversity loss and climate change impacts for each lignocellulose residue type.

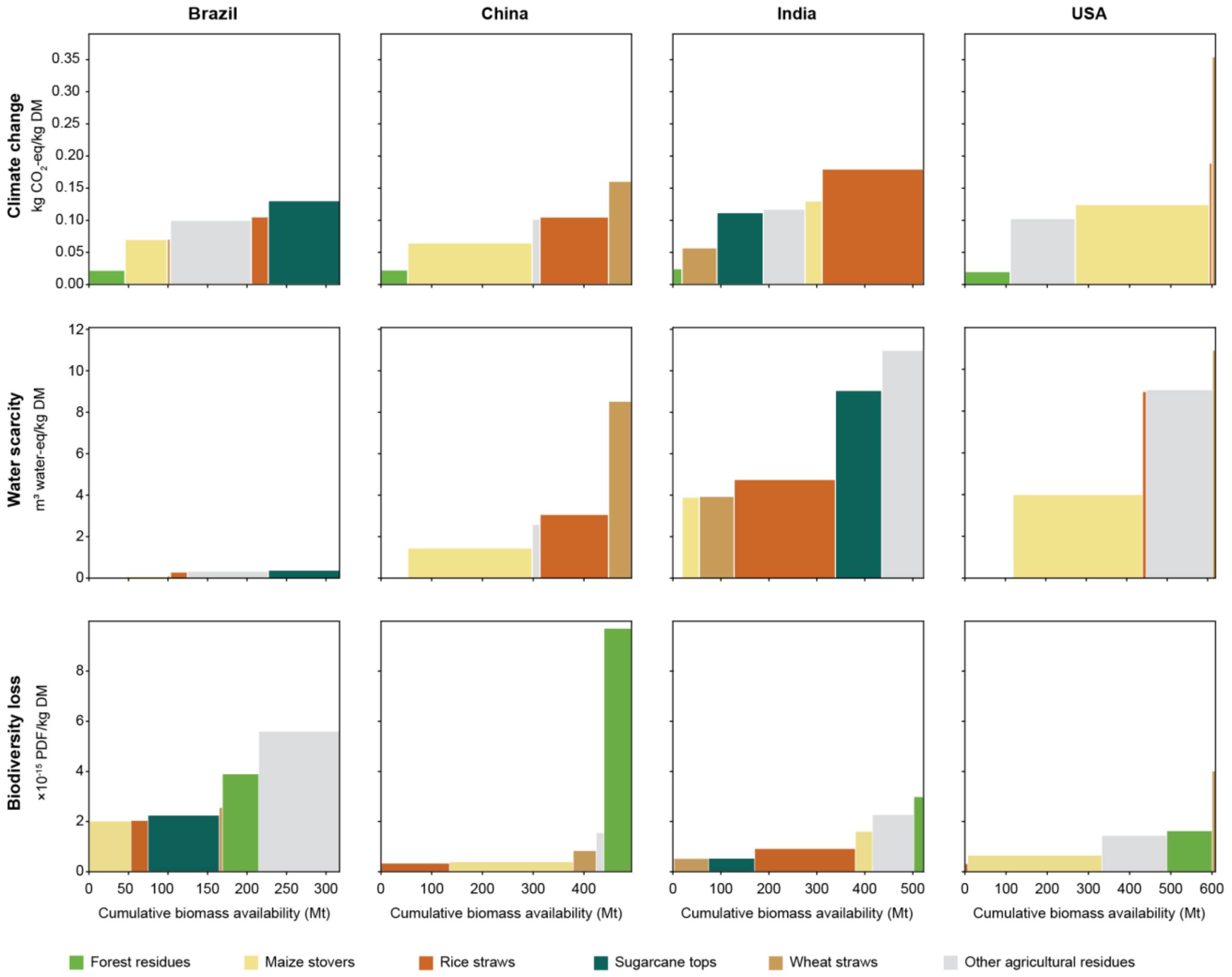

Figure S17. Climate change, water stress, and biodiversity loss merit-order curves of lignocellulose residues in Brazil, China, India, and the United States under the RCP1.9 scenario in 2050.

S2.7. Contribution analysis of climate change impacts of biobased platform chemical

To contextualize our results in a transition towards a sustainable chemical industry, a case study is performed to assess the impacts associated with production of biobased platform chemicals (glucose, xylose, and lignin), considering the RCP1.9 scenario for 2050. The climate change impacts are highlighted in Figure S18, showcasing a range from 1.4 to 2.8 kg CO_2-eq/kg chemical.

As expected, the choice of biomass feedstock plays a key role in determining the climate change impacts of biobased chemical production. While feedstocks from forest residues contribute negligible climate change impacts on global average (scenarios S1, S2, S4, and S5), the use of agricultural residues may account for up to 20% of the overall impact (scenarios S3 and S6).

Low-impact feedstock alone does not guarantee overall low climate change impacts of biobased chemicals. Electricity consumption is another major determinant, where 4.9 kWh is required per kg of chemical products, particularly for solvent recycling. The impacts of electricity vary depending on the carbon intensity of the region's energy mix, typically ranging from 0.10 to 0.97 kg CO_2-eq/kg chemical.

In comparison with chemicals produced with existing technologies, glucose from maize starch has a carbon footprint of 0.69 kg CO_2-eq/kg when the energy mix is updated with premise to represent the RCP1.9 scenario in 2050, as per ecoinvent 3.8 database[31]. However, maize starch is not a focus of this study due to concerns about potential competition with food.

This case study is based on process simulations of a laboratory-scale protocol, emphasizing the need for process optimization prior to industrialization.

In addition, biogenic carbon storage, achieved with durable product design and proper end-of-life management, offers opportunities to reduce the climate change impacts throughout the lifecycle of biobased chemicals.

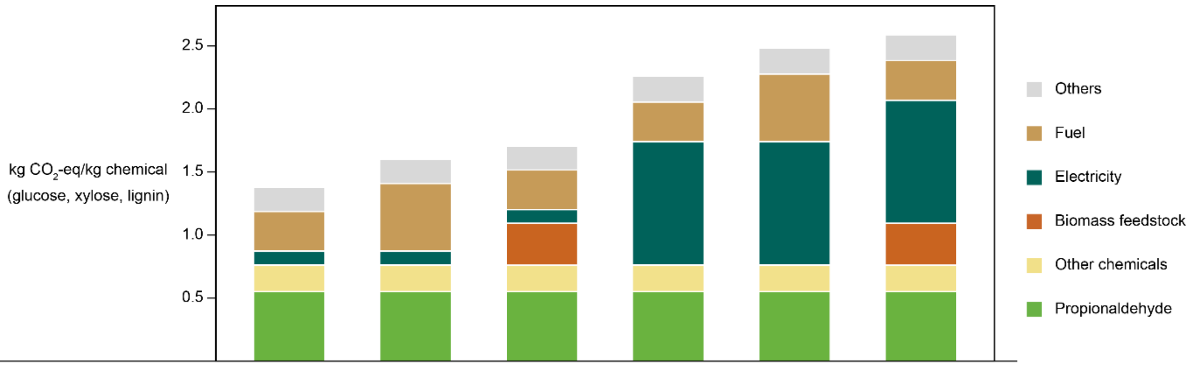

	S1	S2	S3	S4	S5	S6
Biogenic CO_2 impact for fuel considered	No	Yes	No	No	Yes	No
Electricity impact scenario	Min	Min	Min	Max	Max	Max
Biomass feedstock impact scenario	Min	Min	Max	Min	Min	Max

Figure S18. Climate change impacts of biobased platform chemicals based on propionaldehyde fractionation in 2050 under the RCP1.9 scenario.

It reflects the climate change impacts of producing 1 kg biobased platform chemicals (glucose, xylose and lignin) from propionaldehyde fractionation of lignocellulose residues based on mass allocation. Scenario S1–S6 represent different considerations of biogenic CO_2 impacts for fuel (No: GWP_{bio}=0; Yes: GWP_{bio}=0.43, assuming 100-year rotation period), electricity impact scenarios (min: 0.022 kg CO_2-eq/kWh, in Japan; max: 0.198 kg CO_2-eq/kWh,in Russia), and biomass feedstock scenarios (min: 0.004 kg CO_2-eq/kg DM, wood chips in China; max: 0.315 kg CO_2-eq/kg DM, rapeseed straw in Bangladesh).

S3. Uncertainties and sensitivities

S3.1. Sensitivity analysis

S3.1.1. Residues price

We assume that agricultural residues share the same price as forest residues, because they are considered as perfect substitutes for each other in biobased chemicals. However, agricultural residues may be used as animal food and bedding in addition, which may increase its price. If this were the case, more impacts would be allocated to agricultural residues than in this study.

Due to the higher demand, biomass residues have higher price in 2050 under RCP1.9 than under RCPref. On the other hand, the price of the main products (crops and wood products) does not show a big gap between the two RCP scenarios. As a result of economic allocation, more impact is allocated to biomass residues and less impact is allocated to the main product. This partially explains why the climate change impacts of crop residues under RCP1.9 in some countries show a growing trend in the future. We therefore did a sensitivity analysis of the climate change impacts of aggregated crop residues in the major biomass producing countries and assumed the price of biomass residues under RCP1.9 is the same as under RCPref (Figure S19). Under this assumption, the climate change impacts of crop residues under RCP1.9 is lower than RCPref because of the cleaner energy systems.

S3.1.2. GLOBIOM model with endogenous supply of crop residues

Crop residues are not endogenously included in the GLOBIOM model as biomass resource. Instead, GLOBIOM assumes that a constant of 31 EJ biomass is supplied by other biomass (including crop residues) than wood and energy crops. However, when more crop residues are used to satisfy the biomass demand depicted by SSP and RCP scenarios, less energy crops would be needed and hence less land would be transformed for plantation. To understand the impact of crop residues on land use change, a sensitivity analysis was conducted to include crop residues endogenously in the GLOBIOM model under some simplified assumptions:

- 50% of the total crop residues are left on the field for ecological reasons.
- No trade in crop residues
- Heat values and densities are same for all crops: 16 GJ/tonne dry mass and 0.45 tonne dry mass/m^3
- Constant crop residues yields are assumed according to Holmatov et al.[32]

As such, the full availability of crop residues are utilized to satisfy the biomass demand under RCP1.9 (Figure S20). Crop residues and forest residues in total supply 3.5 Gt of biomass in 2050 under RCP1.9. This value falls well in the 3.0–5.2 Gt DM range of the *available* potential in the main study (Figure S20). Compared with the base model, the energy crop demand decreases by 33%. This lead to 41% decrease of land used for plantation globally. However, the total land for plantation only accounts for less than 1% of the global land use. Therefore, the land use in this sensitivity analysis does not show a very different pattern compared with the base model (Figure S21). On the other hand, cropland area is increased slightly globally speaking —4% more than the base case. This indicates crop residues are a more preferred choice of biomass than energy crops.

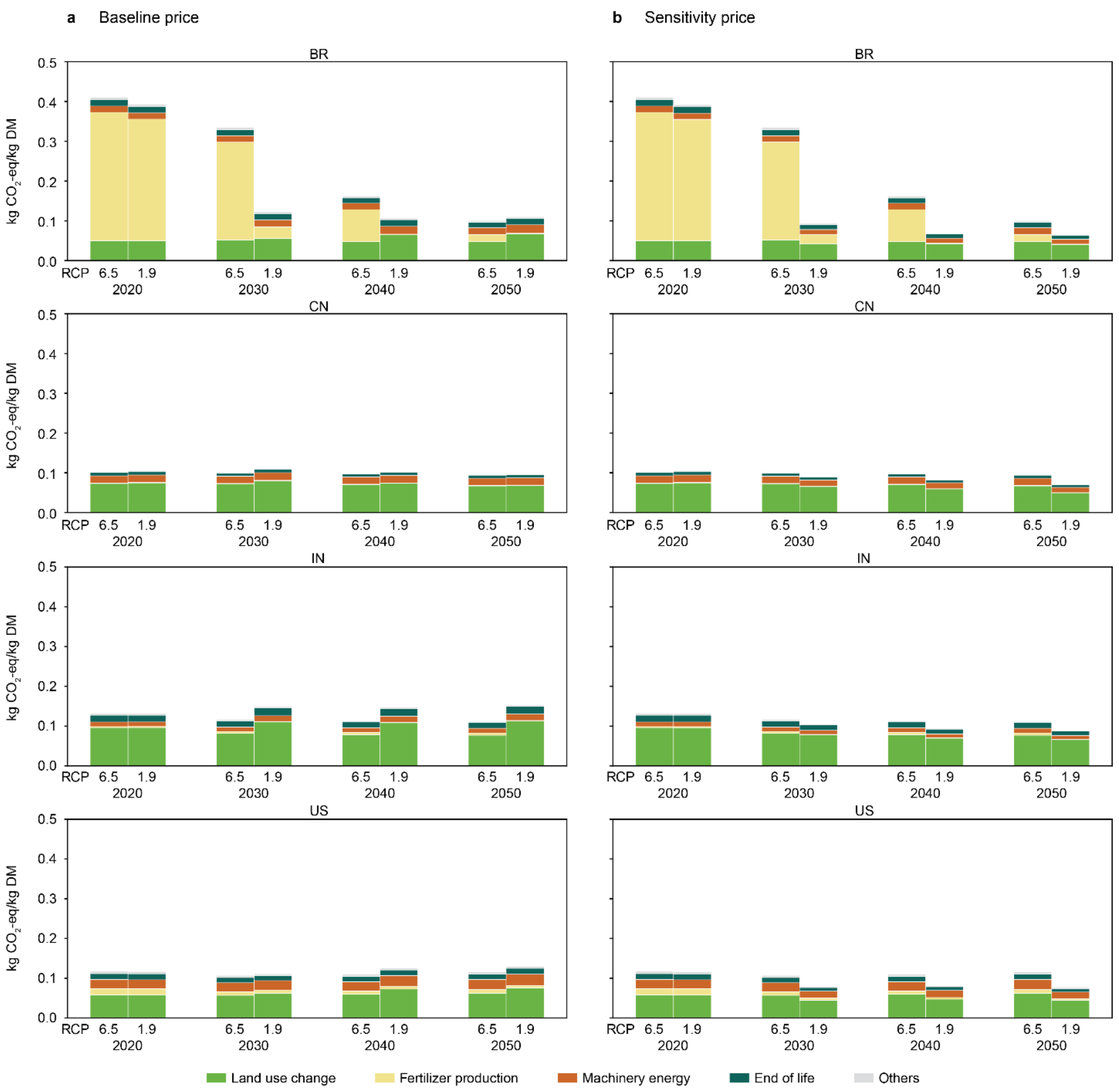

Figure S19. Sensitivity analysis: the impact of biomass residue price on climate change impacts of aggregated crop residues.

a, baseline scenario, the biomass residue price as in GLOBIOM model; b, sensitivity scenario, assuming the price of biomass residues under RCP1.9 is the same as under RCPref

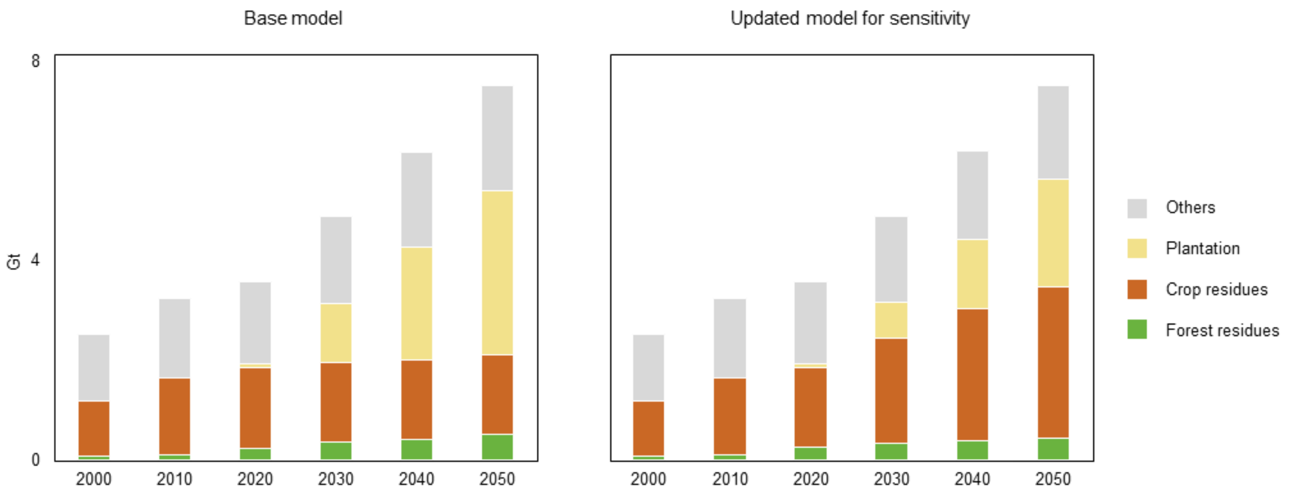

Figure S20. Sensitivity analysis: solid biomass supply under RCP1.9

with the base GLOBIOM model where solid biomass supplied by crop residues is fixed at 31 EJ, and an updated model where crop residues are endogenously considered.

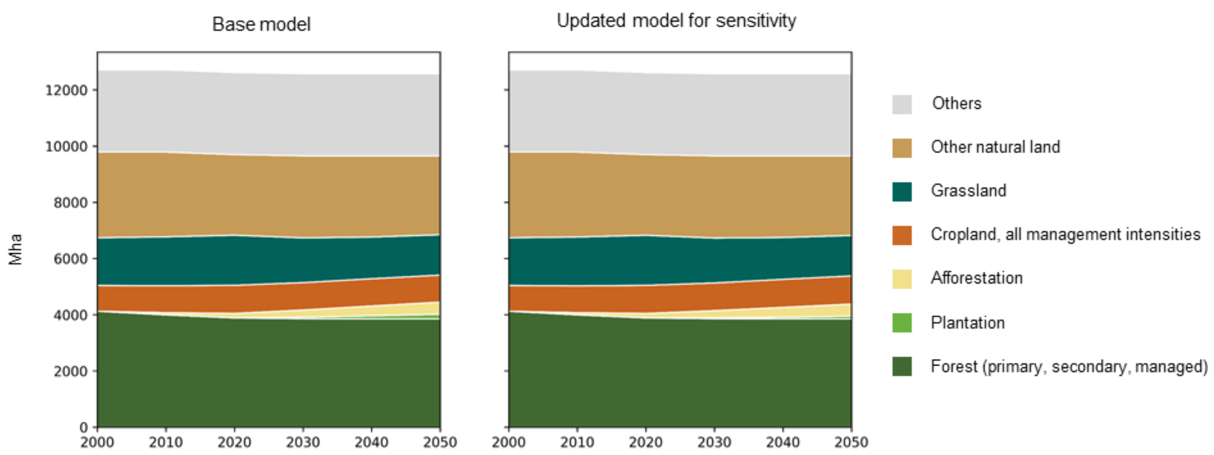

Figure S21. Sensitivity analysis: global land use under RCP1.9

with the base GLOBIOM model where solid bioenergy supplied by crop residues is fixed at 31 EJ, and an updated model where crop residues are endogenously considered.

S3.2. Uncertainties and limitations

S3.2.1. Product demand

The uncertainty of the *available* potential of lignocellulose biomass residues is partially addressed by the choice of different residue-to-crop ratio (RPR) empirical functions to capture the possible range of residue yield. However, the *available* potential of lignocellulose biomass residues is also affected by the production and consumption of crop and wood products, which are highly uncertain for future scenarios. In this study, we examine the future scenarios under the narrative of the Shared Socioeconomic Pathway 2 (SSP2), which represents a moderate development scenario[33]. Yet we do acknowledge that the uncertainty of consumption behavior are depicted in other SSP scenarios. Daioglou et al.[34] projected a global availability of agricultural and forest residues in 2050 based on the IMAGE IAM model[35]. They found that the different SSP scenarios do not have a strong impact on the *available* potential.

S3.2.2. End-of-life biogenic CO_2 emissions

Currently, there is a lack of consensus on the methodology for assessing the climate change impacts of biogenic CO_2 emissions. The uncertainties arise when considering the varied rotation periods and management practices of biomass sources, as well as carbon storage time before CO_2 release. For agricultural residues, with typically short rotation periods, the assumption is that the release of biogenic CO_2 has negligible impact on global warming. In contrast, the impact of biogenic CO_2

emissions from forest residues is more uncertain due to the influence of longer rotation periods and diverse forest management practices. The extent to which these factors affect the net climate impact of biogenic CO_2 emissions is not well established. Furthermore, the climate change impacts of different end-of-life scenarios for biobased products, such as incineration or recycling, add another layer of uncertainty. With recycling, the carbon storage in the biobased products is longer, and the climate change impacts of biogenic CO_2 emissions can be reduced.

S3.3.3. Climate change impacts of intensified forest management

More forests will be under intensified management to satisfy the growing biomass demand under RCP1.9, which may lead to a decrease in carbon stocks in the forest. This decrease can be attributed to several key factors. Firstly, the shift towards monoculture and the prioritization of fast-growing species for economic gain often leads to a homogenization of the forest ecosystem, which can result in lower biomass per area as these species may not store as much carbon as a diverse array of native species. Secondly, intensified practices such as frequent harvesting and soil disturbance from mechanical operations disrupt soil carbon pools, leading to direct releases of carbon dioxide into the atmosphere. Additionally, shorter rotation periods prevent forests from reaching their full carbon storage potential, as mature forests typically hold more carbon in their biomass and soil than young forests. These factors collectively contribute to a reduction in the forest's ability to act as a carbon sink, thus posing the challenge of climate change mitigation. Therefore, forest residues from intensified forests might have higher climate change impacts than from forests under minimal or light management levels. However, the management levels are not differentiated the IPCC guidelines[21], and hence, the climate change impacts of intensified forest management are not quantified in this study.

S3.3.4. Additional environmental impacts caused by lignocellulose residue removal

Our analysis incorporates ecological constraints to ensure that a significant proportion of residues (28-43%) remains on the field, compared to the current global average of 42-49% of cereal residues left in situ.[36] Previous studies suggest that the removal of residues from croplands and forests could potentially impact soil organic carbon (SOC) stocks, which can be a main driver of climate change impacts associated with lignocellulose residue-based products (e.g., biofuels).[37-39] However, globally, up to 26% of all cereal residues (considered within our study's "sustainable potential") are currently used for purposes such as domestic fuel or are burnt on the field.[36] In developing countries, this number is even higher.[36] Redirecting these residues from such uses does not

inherently result in additional SOC loss. Therefore, applying a global emission factor from the literature, which assumes no current residue harvest, would overestimate the climate change impacts associated with SOC changes from lignocellulose residues. In addition, the effects of residue removal on SOC are complex and vary widely by local climate, soil type, and agricultural and forestry practices, making it a challenging impact to quantify accurately on a global scale.

The biodiversity impacts of removing residues, particularly from forests, have not been directly quantified in this study. Residues play a critical role in maintaining ecological functions and biodiversity by providing habitats. Although our analysis maps biodiversity impacts to different types of land use and land use change, it does not specifically distinguish impacts based on the presence or absence of residue removal, which may lead to underestimations of the biodiversity impacts associated with lignocellulose residue-based products.

To better reflect the complexity and regional variability of environmental impacts due to residue removal, future studies should develop more region-specific guidelines for quantifying SOC changes and biodiversity loss impacts due to residue removal, similar to existing IPCC guidelines for land use change.[21] Such guidelines would enhance the accuracy of environmental impact assessments and support more sustainable residue management practices.

S3.3.5. Other uncertainties

The life-cycle assessment in this study relies on background databases such as ecoinvent 3.8 and Agri-footprint 6. These databases include the production of fertilizers and other chemical feedstocks under current technology, which is mostly fossil-based production pathways. As there is no information regarding the future low-carbon production pathways of fertilizers and other chemical feedstocks, these background databases with fossil-based pathways were utilized with updated energy mixes for the future scenarios. This may lead to an overestimation of the climate change impacts, especially for 2050 under RCP1.9 scenario.

The cradle-to-gate climate change impacts of forest residues are mainly determined by the energy use during harvest. We acknowledge the regional differences in wood harvesting methods, however, as such information is not available for most countries, the harvest activities in Switzerland in ecoinvent 3.8 are used for all other countries. Wood harvest in Switzerland is highly mechanized, with even 1.1–3.2% wood being harvested by helicopter[40]. In other countries with less mechanization of wood harvest, this impact would be smaller. Despite the potential over-estimation

of the cradle-to-gate climate change impacts, forest residues still present lower climate change impact than agricultural residues.

S4. References

1. Havlik, P.; Valin, H.; Herrero, M.; Obersteiner, M.; Schmid, E.; Rufino, M. C.; Mosnier, A.; Thornton, P. K.; Bottcher, H.; Conant, R. T.; Frank, S.; Fritz, S.; Fuss, S.; Kraxner, F.; Notenbaert, A., Climate change mitigation through livestock system transitions. *P Natl Acad Sci USA* **2014,** *111* (10), 3709-3714.
2. Havlik, P.; Valin, H.; Mosnier, A.; Obersteiner, M.; Baker, J. S.; Herrero, M.; Rufino, M. C.; Schmid, E., Crop Productivity and the Global Livestock Sector: Implications for Land Use Change and Greenhouse Gas Emissions. *Am J Agr Econ* **2013,** *95* (2), 442-448.
3. Lauri, P.; Havlik, P.; Kindermann, G.; Forsell, N.; Bottcher, H.; Obersteiner, M., Woody biomass energy potential in 2050. *Energ Policy* **2014,** *66*, 19-31.
4. Ronzon, T.; Piotrowski, S., Are primary agricultural residues promising feedstock for the European bioeconomy? *Industrial Biotechnology* **2017,** *13* (3), 113-127.
5. Bentsen, N. S.; Felby, C.; Thorsen, B. J., Agricultural residue production and potentials for energy and materials services. *Prog Energ Combust* **2014,** *40*, 59-73.
6. Scarlat, N.; Martinov, M.; Dallemand, J. F., Assessment of the availability of agricultural crop residues in the European Union: Potential and limitations for bioenergy use. *Waste Manage* **2010,** *30* (10), 1889-1897.
7. Abbas, A.; Ansumali, S., Global Potential of Rice Husk as a Renewable Feedstock for Ethanol Biofuel Production. *Bioenerg Res* **2010,** *3* (4), 328-334.
8. Tolessa, A.; Bélières, J. F.; Salgado, P.; Raharimalala, S.; Louw, T. M.; Goosen, N. J., Assessment of Agricultural Biomass Residues for Anaerobic Digestion in Rural Vakinankaratra Region of Madagascar. *Bioenerg Res* **2022,** *15* (2), 1251-1264.
9. Jenjariyakosoln, S.; Gheewala, S. H.; Sajjakulnukit, B.; Garivait, S., Energy and GHG emission reduction potential of power generation from sugarcane residues in Thailand. *Energy Sustain Dev* **2014,** *23*, 32-45.
10. Lauri, P.; Forsell, N.; Gusti, M.; Korosuo, A.; Havlik, P.; Obersteiner, M., Global Woody Biomass Harvest Volumes and Forest Area Use Under Different SSP-RCP Scenarios. *J Forest Econ* **2019,** *34* (3-4), 285-309.
11. IPCC *Good Practice Guidance for Land Use, Land-Use Change and Forestry*; The Intergovernmental Panel on Climate Change: Hayama, Japan, 2003.
12. OECD *Global Plastics Outlook: Economic Drivers, Environmental Impacts and Policy Options*; OECD Publishing: Paris, France, 2022.
13. Meys, R.; Katelhon, A.; Bachmann, M.; Winter, B.; Zibunas, C.; Suh, S.; Bardow, A., Achieving net-zero greenhouse gas emission plastics by a circular carbon economy. *Science* **2021,** *374* (6563), 71-+.
14. Mutel, C., Brightway: An open source framework for Life Cycle Assessment. *Journal of Open Source Software* **2017,** *2* (12), 236.
15. Lauri, P.; Forsell, N.; Di Fulvio, F.; Snall, T.; Havlik, P., Material substitution between coniferous, non-coniferous and recycled biomass - Impacts on forest industry raw material use and regional competitiveness. *Forest Policy Econ* **2021,** *132*.
16. BSI *PAS 2050-1 Assessment of life cycle greenhouse gas emissions from horticultural products*; The British Standards Institution: London, UK, 2012.
17. Pfister, S.; Bayer, P.; Koehler, A.; Hellweg, S., Environmental Impacts of Water Use in Global Crop Production: Hotspots and Trade-Offs with Land Use. *Environ Sci Technol* **2011,** *45* (13), 5761-5768.
18. Ludemann, C. I.; Gruere, A.; Heffer, P.; Dobermann, A., Global data on fertilizer use by crop and by country. *Sci Data* **2022,** *9* (1).

19. FAO, FAOSTAT database: Fertilizers by Product. July 15, 2022 ed.; Food and Agriculture Organization of the United Nations: Rome, Italy, 2023.
20. Blonk, H.; Tyszler, M.; Paassen, M. v.; Braconi, N.; Draijer, N.; Rijn, J. v. *Agri-footprint 6 methodology report. Part 2: description of data*; Blonk: Gouda, the Netherlands, 2022.
21. IPCC *2006 IPCC Guidelines for National Greenhouse Gas Inventories - Volume 4 - Agriculture, Forestry and Other Land Use* Intergovernmental Panel on Climate Change: Kanagawa, Japan, 2006.
22. FAO *Global Forest Resources Assessment 2020*; Food and Agriculture Organization of the United Nations: Rome, Italy, 2020.
23. Fischer, G.; Nachtergaele, F.; Prieler, S.; van Velthuizen, H.; Verelst, L. *Global Agro-ecological Zones Assessment for Agriculture (GAEZ 2008)*; IIASA, Laxenburg, Austria: and FAO, Rome, Italy, 2008.
24. IPCC *2019 Refinement to the 2006 IPCC Guidelines for National Greenhouse Gas Inventories - Volume 4 - Agriculture, Forestry and Other Land Use* Intergovernmental Panel on Climate Change: Geneva, Switzerland, 2019.
25. Scherer, L.; De Laurentiis, V.; Marques, A.; Michelsen, O.; Alejandre, E. M.; Pfister, S.; Rosa, F.; Rugani, B., Linking land use inventories to biodiversity impact assessment methods COMMENT. *Int J Life Cycle Ass* **2021,** *26* (12), 2315-2320.
26. Amiri, M. T.; Dick, G. R.; Questell-Santiago, Y. M.; Luterbacher, J. S., Fractionation of lignocellulosic biomass to produce uncondensed aldehyde-stabilized lignin. *Nat Protoc* **2019,** *14* (3), 921-954.
27. Wooley, R. J.; Putsche, V. *Development of an ASPEN PLUS Physical Property Database for Biofuels Components*; National Renewable Energy Laboratory: Colorado, the U.S., 1996.
28. IHS Markit *Process Economics Program (PEP) Yearbook*; 2021.
29. Bazzanella, A. M.; Ausfelder, F. *Low carbon energy and feedstock for the European chemical industry*; DECHEMA: Frankfurt am Main, Germany, 2017.
30. Hoppe, W.; Thonemann, N.; Bringezu, S., Life Cycle Assessment of Carbon Dioxide-Based Production of Methane and Methanol and Derived Polymers. *J Ind Ecol* **2018,** *22* (2), 327-340.
31. Wernet, G.; Bauer, C.; Steubing, B.; Reinhard, J.; Moreno-Ruiz, E.; Weidema, B., The ecoinvent database version 3 (part I): overview and methodology. *Int J Life Cycle Ass* **2016,** *21* (9), 1218-1230.
32. Holmatov, B.; Schyns, J. F.; Krol, M. S.; Gerbens-Leenes, P. W.; Hoekstra, A. Y., Can crop residues provide fuel for future transport? Limited global residue bioethanol potentials and large associated land, water and carbon footprints. *Renew Sust Energ Rev* **2021,** *149*.
33. Riahi, K.; van Vuuren, D. P.; Kriegler, E.; Edmonds, J.; O'Neill, B. C.; Fujimori, S.; Bauer, N.; Calvin, K.; Dellink, R.; Fricko, O.; Lutz, W.; Popp, A.; Cuaresma, J. C.; Samir, K. C.; Leimbach, M.; Jiang, L. W.; Kram, T.; Rao, S.; Emmerling, J.; Ebi, K.; Hasegawa, T.; Havlik, P.; Humpenoder, F.; da Silva, L. A.; Smith, S.; Stehfest, E.; Bosetti, V.; Eom, J.; Gernaat, D.; Masui, T.; Rogelj, J.; Strefler, J.; Drouet, L.; Krey, V.; Luderer, G.; Harmsen, M.; Takahashi, K.; Baumstark, L.; Doelman, J. C.; Kainuma, M.; Klimont, Z.; Marangoni, G.; Lotze-Campen, H.; Obersteiner, M.; Tabeau, A.; Tavoni, M., The Shared Socioeconomic Pathways and their energy, land use, and greenhouse gas emissions implications: An overview. *Global Environ Chang* **2017,** *42*, 153-168.

34. Daioglou, V.; Stehfest, E.; Wicke, B.; Faaij, A.; van Vuuren, D. P., Projections of the availability and cost of residues from agriculture and forestry. *Gcb Bioenergy* **2016,** *8* (2), 456-470.
35. Friedl, A.; Padouvas, E.; Rotter, H.; Varmuza, K., Prediction of heating values of biomass fuel from elemental composition. *Anal Chim Acta* **2005,** *544* (1-2), 191-198.
36. Smerald, A.; Rahimi, J.; Scheer, C., A global dataset for the production and usage of cereal residues in the period 1997-2021. *Sci Data* **2023,** *10* (1).
37. Kim, S.; Zhang, X. S.; Dale, B.; Reddy, A. D.; Jones, C. D.; Cronin, K.; Izaurralde, R. C.; Runge, T.; Sharara, M., Corn stover cannot simultaneously meet both the volume and GHG reduction requirements of the renewable fuel standard. *Biofuel Bioprod Bior* **2018,** *12* (2), 203-212.
38. Lan, K.; Zhang, B. Q.; Lee, T.; Yao, Y., Soil organic carbon change can reduce the climate benefits of biofuel produced from forest residues. *Joule* **2024,** *8* (2).
39. Liska, A. J.; Yang, H. S.; Milner, M.; Goddard, S.; Blanco-Canqui, H.; Pelton, M. P.; Fang, X. X.; Zhu, H. T.; Suyker, A. E., Biofuels from crop residue can reduce soil carbon and increase CO_2 emissions. *Nat Clim Change* **2014,** *4* (5), 398-401.
40. ecoinvent *ecoinvent 3.8 Dataset Documentation*; Zurich, Switzerland, 2021.
